# NK Cells Stimulate Recruitment of cDC1 into the Tumor Microenvironment Promoting Cancer Immune Control

**DOI:** 10.1016/j.cell.2018.01.004

**Published:** 2018-02-22

**Authors:** Jan P. Böttcher, Eduardo Bonavita, Probir Chakravarty, Hanna Blees, Mar Cabeza-Cabrerizo, Stefano Sammicheli, Neil C. Rogers, Erik Sahai, Santiago Zelenay, Caetano Reis e Sousa

**Affiliations:** 1 Immunobiology Laboratory, The Francis Crick Institute, 1 Midland Road, London NW1 1AT, UK; 2 Cancer Inflammation and Immunity Group, CRUK Manchester Institute, The University of Manchester, Manchester M20 4BX, UK; 3 Bioinformatics, The Francis Crick Institute, 1 Midland Road, London NW1 1AT, UK; 4 Tumour Cell Biology Laboratory, The Francis Crick Institute, 1 Midland Road, London NW1 1AT, UK

**Keywords:** dendritic cells, tumor microenvironment, tumor immune control, cancer immunotherapy, immune evasion

## Abstract

Conventional type 1 dendritic cells (cDC1) are critical for antitumor immunity, and their abundance within tumors is associated with immune-mediated rejection and the success of immunotherapy. Here, we show that cDC1 accumulation in mouse tumors often depends on natural killer (NK) cells that produce the cDC1 chemoattractants CCL5 and XCL1. Similarly, in human cancers, intratumoral CCL5, XCL1, and XCL2 transcripts closely correlate with gene signatures of both NK cells and cDC1 and are associated with increased overall patient survival. Notably, tumor production of prostaglandin E2 (PGE_2_) leads to evasion of the NK cell-cDC1 axis in part by impairing NK cell viability and chemokine production, as well as by causing downregulation of chemokine receptor expression in cDC1. Our findings reveal a cellular and molecular checkpoint for intratumoral cDC1 recruitment that is targeted by tumor-derived PGE_2_ for immune evasion and that could be exploited for cancer therapy.

## Highlights

• NK cells recruit cDC1 into the tumor microenvironment
• cDC1 recruitment depends on NK cell-derived chemokines CCL5 and XCL1
• The NK cell/chemokine/cDC1 axis is associated with cancer patient survival
• Tumor-derived PGE_2_ impairs NK cell and cDC1 resulting in cancer immune evasion

## Graphical Abstract

**Figure:**
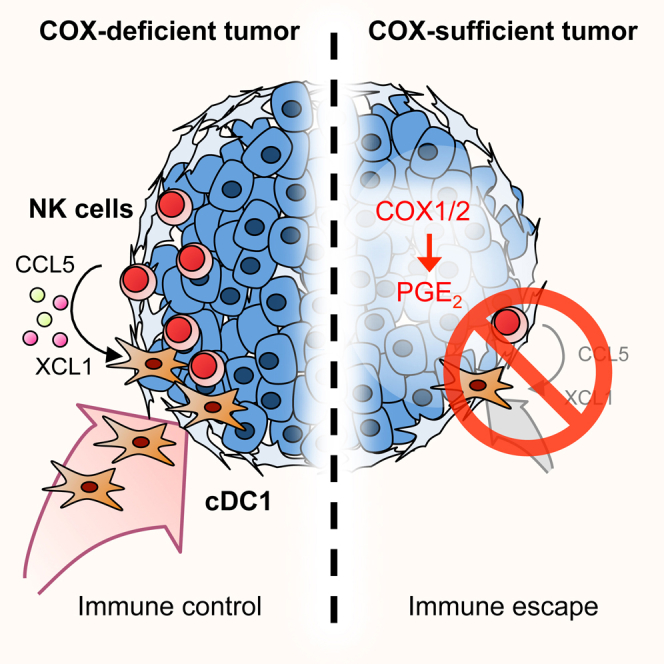

## Teaser

Natural killer cells recruit dendritic cells to the tumor microenvironment, and disruption of this process results in cancer immune evasion.

## Introduction

The tumor microenvironment (TME) contains stromal cells and immune cells that shape cancer development and impact the response to tumor therapy (Hanahan and Weinberg, 2011, Palucka and Coussens, 2016). Intratumoral immune cells comprise lymphocytes, such as T cells, B, cells and natural killer (NK) cells, and diverse populations of myeloid cells, including granulocytes, monocytes, macrophages, and dendritic cells (DCs) (Gajewski et al., 2013, Hanahan and Weinberg, 2011, Palucka and Coussens, 2016). The different myeloid cells within the TME fulfill distinct and sometimes opposing roles. Simplistically, intratumoral monocytes and M2-polarized macrophages can promote cancer cell growth, angiogenesis, and metastasis, as well as contribute to the establishment of an immunosuppressive environment. They are associated with tumor progression and poor clinical outcome (Noy and Pollard, 2014). In contrast, M1-polarized macrophages and DCs contribute to anti-tumor immunity and are associated with a favorable outcome (Engblom et al., 2016).

The contribution of conventional DCs (cDCs) to anti-tumor immunity reflects their ability to present tumor antigens and to secrete cytokines that regulate T cell survival and effector function. cDCs can be divided into at least two subsets, conventional type 1 dendritic cells (cDC1) and conventional type 2 dendritic cells (cDC2) (Guilliams et al., 2014, Merad et al., 2013). The cDC1 subset depends for its development on the transcription factor Batf3 and can be identified by the selective expression of the C-type lectin receptor DNGR-1 (aka CLEC9A) and the chemokine receptor XCR1 and, in non-lymphoid organs and in tumors, additional expression of the integrin αE (CD103) in the presence of low expression of CD11b. cDC1 are especially adept at taking up dead tumor cells and transporting tumor antigens to tumor-draining lymph nodes where they constitute the key DC subtype responsible for cross-priming anti-tumor CD8^+^ T cells (Roberts et al., 2016, Salmon et al., 2016). In addition to this trafficking role, cDC1 also play a key role within tumors themselves. Intratumoral cDC1 attract T cells (Spranger et al., 2017), re-stimulate and expand tumor-specific CD8^+^ T cells (Broz et al., 2014), and support T cell effector function by secreting interleukin (IL)-12 (Ruffell et al., 2014). The overall importance of cDC1 in anti-tumor immunity is underscored by multiple studies demonstrating that the lack of cDC1 in *Batf3*^−/−^ mice abolishes the rejection of immunogenic tumors and the response to adoptive T cell therapy and to immune checkpoint blockade (Broz et al., 2014, Hildner et al., 2008, Salmon et al., 2016, Sánchez-Paulete et al., 2016, Spranger et al., 2015).

Human cDC1 are very rare within the TME and often excluded from early tumor stages, which might hinder anti-tumor immunity and contribute to cancer progression. Although intratumoral cDC1 have not been investigated in humans in as much detail as in mice, cDC1 abundance in human melanoma correlates with T cell infiltration and the ratio of cDC1-selective transcripts over macrophage-restricted transcripts can be used as a prognostic marker for cancer patient survival (Broz et al., 2014, Spranger et al., 2017). Therapies aimed at increasing cDC1 abundance in tumors or facilitating their activation may therefore boost anti-tumor immunity and potentially increase the responsiveness of cancer patients to immunotherapy (Broz et al., 2014, Salmon et al., 2016, Spranger et al., 2017). However, the mechanisms determining the abundance of cDC1 at the tumor site remain enigmatic and it is unclear whether cDC1 are actively recruited into the TME and if this requires the participation of other cell types.

Prostaglandin E2 (PGE_2_) is a prostanoid with immune-regulatory function that is produced by many cell types and can further be released upon cell death (Hangai et al., 2016). We previously found that many tumors secrete PGE_2_ to suppress anti-cancer immunity (Zelenay et al., 2015). In such tumors, genetic ablation of cyclooxygenases, encoded by the *Ptgs1* and *Ptgs2* genes, leads to inability to produce PGE_2_ and renders the cancers susceptible to cDC1-dependent CD8^+^ T cell-mediated immune control (Zelenay et al., 2015). Mouse tumors lacking PGE_2_ production are therefore an ideal system in which to dissect the mechanisms underlying cDC1 accumulation. Here, we show that such tumors are infiltrated by cDC1, and we identify a key role for intratumoral NK cells in producing CCL5 and XCL1 chemokines that promote cDC1 recruitment. We provide evidence that a similar NK cell/chemokine functional axis determines cDC1 abundance in human melanoma, breast cancer, lung cancer, and head and neck squamous cell carcinoma and show that it impacts on patient survival. Finally, we uncover a role for PGE_2_ both in diminishing NK cell survival and function and in downregulating cDC1 responsiveness to chemoattractants. These data provide insights into the control of cDC1 accumulation in tumors in mice and humans and support the rational design of therapies aiming to increase cDC1 numbers in tumors that might help overcoming resistance to current immunotherapies.

## Results

### cDC1 Accumulate within the Tumor Microenvironment of COX-Deficient Tumors

**Figure 1:**
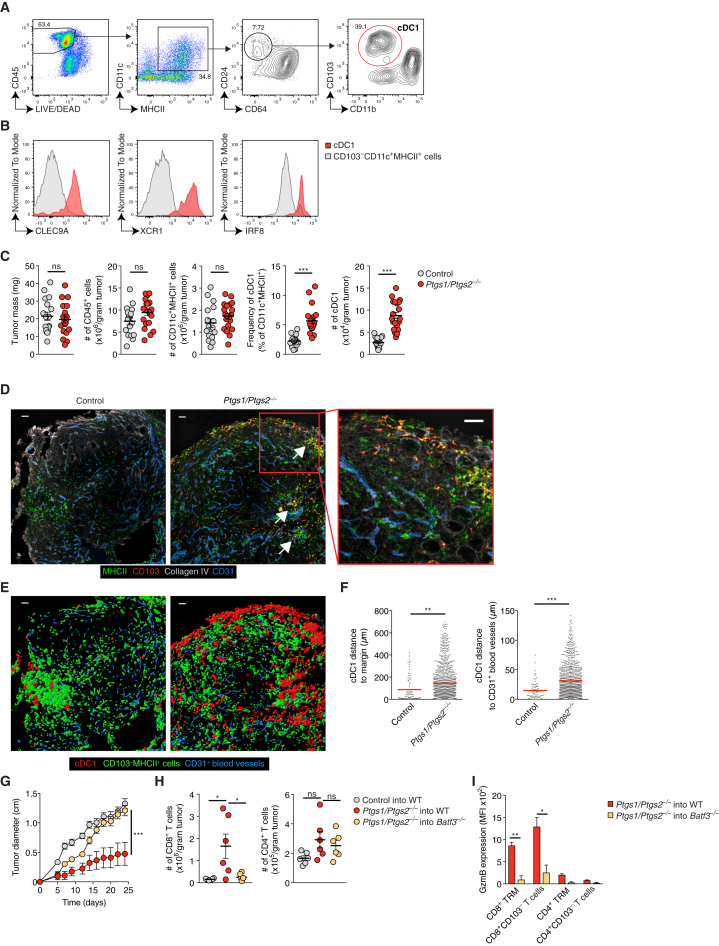
cDC1 Accumulate in the TME of PGE_2_-Deficient Tumors (A and B) Wild-type (WT) mice were injected s.c. with 2 × 10^6^*Ptgs1/Ptgs2*^−/−^ BRAF^V600E^ cells and tumor-infiltrating immune cells were analyzed 4 days later by flow cytometry. Plots representative of 2-3 experiments are shown. (A) Gating strategy to identify intratumoral cDC1. Numbers represent % cells within depicted gate. (B) Analysis of intratumoral cDC1 and CD103^−^CD11c^+^MHCII^+^ cells for cDC1 markers. (C) Quantification of tumor mass and intratumoral immune cells 4 days after inoculation of WT mice with 2 × 10^6^ control or *Ptgs1/Ptgs2*^−/−^ BRAF^V600E^ melanoma cells. Data are pooled from 4 independent experiments with 4–6 mice per group and depicted as mean ± SEM; each circle represents an individual tumor. (D) Representative sections of control and *Ptgs1/Ptgs2*^−/−^ BRAF^V600E^ tumors. Arrows indicate multicellular clusters of cDC1. Scale bar, 50 μm. (E) Surface reconstruction of images from (D) showing the localization of cDC1 versus CD103^−^MHCII^+^ cells. (F) Distance analyses based on (E). Data represent quantification across 8 images from 6 tumors. (G) WT or *Batf3*^−/−^ mice were inoculated with 2 × 10^5^ control or *Ptgs1/Ptgs2*^−/−^ BRAF^V600E^ cells and tumor growth was analyzed over time. Data are represented as mean ± SEM and are from one of two independent experiments with 3–5 mice per group. (H and I) WT and *Batf3*^−/−^ mice were injected s.c. with 2 × 10^6^ control or *Ptgs1/Ptgs2*^−/−^ BRAF^V600E^ cells. 12 days later, T cells were quantified (H) and stained for intracellular GzmB (I). In (C) and (F)–(I): n.s., non-significant, ^∗^p < 0.05, ^∗∗^p < 0.0, ^∗∗∗^p < 0.001. See also Figure S1.

**Figure S1:**
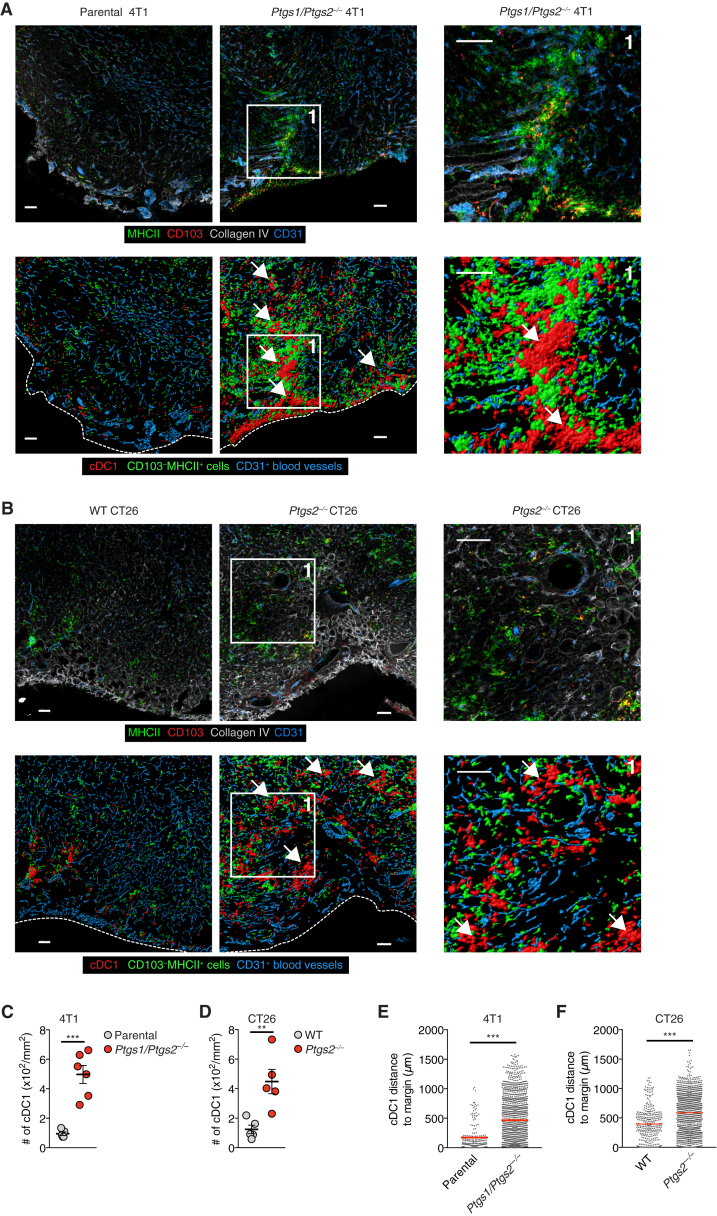
Accumulation and Positioning of cDC1 in COX-Deficient Tumors, Related to Figure 1 WT mice were injected s.c. with 2x10^6^ 4T1 breast cancer cells, CT26 colorectal cancer cells or BRAF^V600E^ melanoma cells. Tumors were excised 4 days later. (A and B) Immunofluorescence images of parental 4T1 or *Ptgs1/Ptgs2*^*−/−*^ 4T1 tumors (A) or WT CT26 or *Ptgs2*^*−/−*^ CT26 tumors (B). Upper panels show original images, lower panels show visualization of CD103^+^ cDC1 localization by surface reconstruction. Scale bar 100μm. Images are representative of individual tumors from 5-6 mice in two independent experiments. The dashed lines indicate the tumor margin, arrows indicate multicellular clusters of cDC1. (C and D) Quantification of intratumoral cDC1 in immunofluorescent images of 4T1 tumors (C) or CT26 tumors (D). Each circle represents data from one individual tumor. Data are mean ± SEM and were pooled from two independent experiments. (E) Distance analysis based on (A). (F) Distance analysis based on (B). Line indicates mean value, ^∗∗^p < 0.01, ^∗∗∗^p < 0.001.

We established a flow cytometry staining protocol that allows distinction between cDC1 and other CD11c^+^MHC class II (MHCII)^+^ myeloid cell populations including CD64^+^ macrophages and CD11b^+^ cDC2 in tumors (Figure 1A). CD103^+^ but not other cells (putative cDC2) among CD64^*−*^CD11c^+^MHCII^+^ cells expressed DNGR-1 (Sancho et al., 2008), XCR1 (Dorner et al., 2009), and IRF8 (Ginhoux et al., 2009) (Figure 1B), validating them as bona fide cDC1. We used the staining protocol to assess cDC1 abundance in PGE_2_-producing control (COX-competent) BRAF^V600E^ and *Ptgs1/Ptgs2*^−/−^ (COX-deficient) BRAF^V600E^ melanoma tumors (Zelenay et al., 2015). We focused initial analyses on 4 days after tumor cell implantation, before the onset of any T cell-mediated immune control of the COX-deficient tumors (Zelenay et al., 2015). As reported (Zelenay et al., 2015), control and *Ptgs1/Ptgs2*^−/−^ BRAF^V600E^ tumors were broadly equivalent in terms of total number of CD45^+^ cells, CD11c^+^MHCII^+^ cells, and tumor mass (Figure 1C). However, *Ptgs1/Ptgs2*^−/−^ BRAF^V600E^ showed markedly greater accumulation of cDC1, both in frequency and in total numbers (Figure 1C) (Zelenay et al., 2015). Consistent with the flow cytometric analysis, cDC1 were sparse in confocal images of sections from control BRAF^V600E^ tumors but abundant in those from *Ptgs1/Ptgs2*^−/−^ BRAF^V600E^ tumors (Figures 1D and 1E). In addition to increased density, cDC1 often formed multicellular clusters within *Ptgs1/Ptgs2*^−/−^ BRAF^V600E^ tumor tissue (Figures 1D and 1E). Tumor infiltration by cDC1s was confirmed by distance analyses after surface reconstruction of cDC1 profiles in confocal images, which revealed that cDC1 in *Ptgs1/Ptgs2*^−/−^ BRAF^V600E^ melanomas were located further away from the tumor margin and from CD31^+^ blood vessels than in control tumors (Figure 1F). We extended the analysis to other mouse cancer models and found that, similar to BRAF^V600E^ melanoma, tumors formed by COX-competent but not COX-deficient CT26 colorectal cancer cells or 4T1 breast cancer cells displayed low numbers of intratumoral cDC1 (Figures S1A–S1D). Again, we observed altered localization of cDC1 in the COX-sufficient tumors, which displayed fewer clusters of cDC1 deep within the tumor parenchyma (Figures S1A–S1F). Finally, we confirmed that cDC1 recruitment is functionally relevant by demonstrating that immune control of COX-deficient tumors is lost in cDC1-deficient *Batf3*^−/−^ mice (Figure 1G) (Zelenay et al., 2015). This loss of control correlated with markedly reduced tumor infiltration by CD8^+^ T cells (Figure 1H) and the fact that the few infiltrating CD8^+^ T cells, including tissue-resident memory T cells (TRM) identified by CD103 expression, did not express granzyme B (GzmB) (Figure 1I). We conclude that tumor-derived PGE_2_ impairs the accumulation and spatial positioning of cDC1 within the TME and that an unknown mechanism induces the accumulation of cDC1 in COX-deficient tumors, which is key for subsequent CD8^+^ T cell-mediated anti-cancer immunity.

### cDC1 Accumulation in COX-Deficient BRAF^V600E^ Melanoma Depends on NK Cells

**Figure 2:**
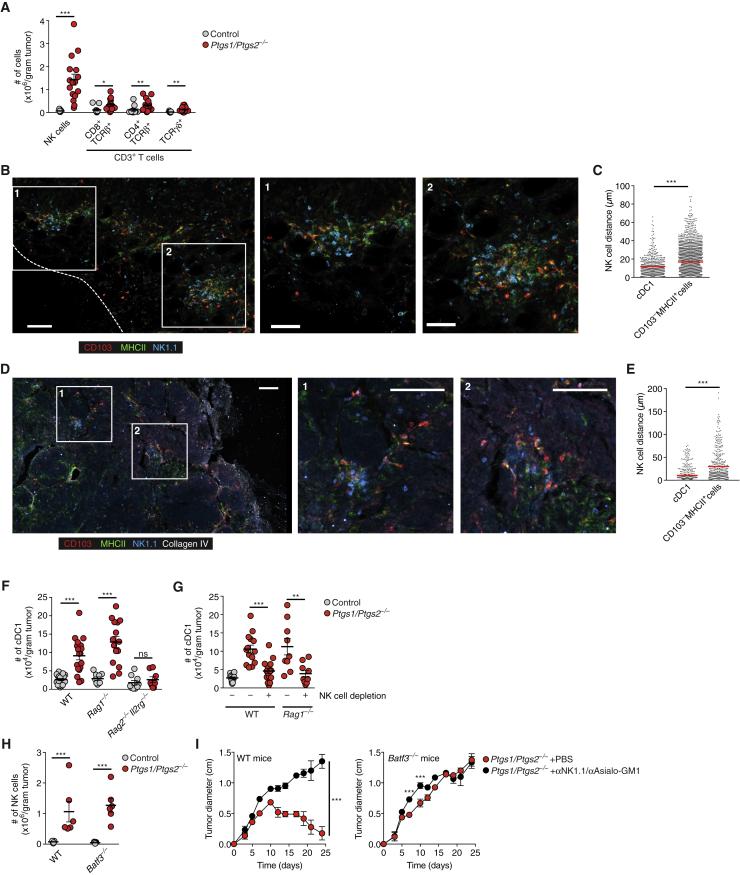
Intratumoral cDC1 Accumulation Depends on NK Cells (A) Quantification of tumor-infiltrating lymphocytes in control or *Ptgs1/Ptgs2*^−/−^ BRAF^V600E^ tumors (day 4). (B) Image of a *Ptgs1/Ptgs2*^−/−^ BRAF^V600E^ tumor. Insets show colocalization of CD103^+^ cDC1 and NK1.1^+^ cells in multicellular clusters. Scale bar, 50 μm. The dashed line indicates the tumor margin. Data are representative of 6 tumors from two experiments. (C) Distance analysis based on (B). Data represent quantification across 6 images from 6 tumors. (D) Image of a tumor from a MMTV-PyMT mouse. Insets show colocalization of CD103^+^ cDC1 and NK1.1^+^ cells. Scale bar, 100 μm. Data are representative of 4 independent experiments. (E) Distance analysis based on (D). Data represent quantification across 8 images from 4 tumors. (F and G) Quantification of cDC1 in control or *Ptgs1/Ptgs2*^−/−^ BRAF^V600E^ tumors 4 days after s.c. inoculation of 2 × 10^6^ tumor cells into WT mice, *Rag1*^−/−^ mice, or *Rag2*^−/−^*Il2rg*^−/−^ mice with or without NK cell depletion. (H) Quantification of NK cells in control or *Ptgs1/Ptgs2*^−/−^ BRAF^V600E^ tumors 4 days after inoculation of WT mice or *Batf3*^−/−^ mice. (I) Effect of NK cell depletion on the growth of *Ptgs1/Ptgs2*^−/−^ BRAF^V600E^ tumors in WT or *Batf3*^−/−^ mice. Data shown in (A) and (F)–(I) are pooled from at least two independent experiments with 4–6 mice per group and represented as mean ± SEM; (A), (C), and (E–I): n.s., non-significant, ^∗^p < 0.05, ^∗∗^p < 0.01, ^∗∗∗^p < 0.001. See also Figure S2.

**Figure S2:**
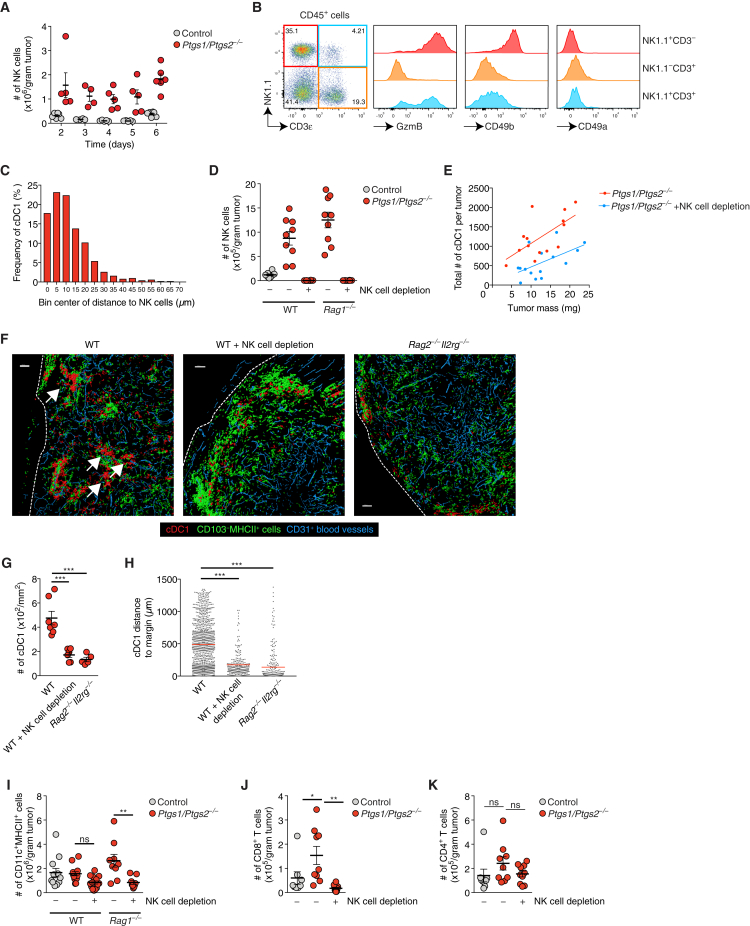
Intratumoral cDC1 Accumulation Depends on NK Cells, Related to Figure 2 (A) Quantification of intratumoral NK cells over time. Each circle represents data for one single BRAF^V600E^ tumor from a group of 4-6 tumors per type and time point. (B) Flow cytometric analysis of intratumoral lymphocytes in a *Ptgs1/Ptgs2*^*−/−*^ BRAF^V600E^ tumor. Data are representative of three independent experiments. (C) Frequency distribution showing the distance of cDC1 to NK1.1^+^ cells within an immunofluorescence image of a *Ptgs1/Ptgs2*^*−/−*^ BRAF^V600E^ tumor. (D) Quantification of intratumoral NK cells after NK cell depletion in the indicated mice given control or *Ptgs1/Ptgs2*^*−/−*^ BRAF^V600E^ tumors. (E) Correlation of total cDC1 numbers and tumor mass in *Ptgs1/Ptgs2*^*−/−*^ BRAF^V600E^ tumors in WT mice or WT mice that were depleted of NK cells prior to tumor cell inoculation. (F) Visualization of CD103^+^ cDC1 localization after surface reconstruction from immunofluorescence images for *Ptgs1/Ptgs2*^*−/−*^ BRAF^V600E^ tumors 4 days after transplantation into WT mice, WT mice depleted of NK cells or *Rag2*^*−/−*^*Il2rg*^*−/−*^mice. Scale bar 100μm. Images are representative of individual tumors from 5-7 mice. Arrows indicate multicellular clusters of cDC1, tumor margins are indicated by dashed lines. (G) Quantification of intratumoral cDC1 in immunofluorescence images of *Ptgs1/Ptgs2*^*−/−*^ BRAF^V600E^ tumors transplanted into WT mice, WT mice that were depleted of NK cells prior to tumor cell inoculation or *Rag2*^*−/−*^*Il2rg*^*−/−*^mice. Each circle represents data for one individual tumor. (H) Distance analyses based on surface reconstruction shown in (F). (I) Quantification of total CD11c^+^MHCII^+^ cells after treatment as in (D). (J) Quantification of intratumoral CD8^+^ T cells after NK cell depletion. (K) Quantification of intratumoral CD4^+^ T cells after NK cell depletion. Analyses shown in B-K were performed 4 days after tumor cell inoculation. Data shown in A, D, E, G and I-K are pooled from at least two independent experiments and represented as mean of all mice in each group ± SEM n.s., non-significant, ^∗^p < 0.05, ^∗∗^p < 0.01, ^∗∗∗^p < 0.001.

In addition to an increase in cDC1 and modest elevation of T cell populations, *Ptgs1/Ptgs2*^−/−^ BRAF^V600E^ melanomas showed a prominent early rise in NK1.1^+^CD3^−^ cells (Figure 2A), which was sustained over several days (Figure S2A). NK1.1^+^CD3^−^ cells stained positive for CD49b and GzmB, suggesting that they were conventional NK cells and not ILC1 (Serafini et al., 2015) (Figure S2B). The distribution of intratumoral NK cells was highly similar to that of cDC1, evident as multicellular clusters of both cell types (Figure 2B) located within 5–10 μm of each other (Figure S2C) and with NK cells positioned closer to cDC1 than to other MHCII^+^ cells (Figure 2C). Similarly, in an autochthonous genetically engineered breast cancer model (MMTV-PyMT), cDC1 and NK cells were often found in multicellular clusters (Figures 2D and 2E), indicating that co-localization is not a consequence of tumor cell transplantation.

Given the close apposition, we tested the requirement of NK cells for cDC1 accumulation by measuring cDC1 content in *Ptgs1/Ptgs2*^−/−^ BRAF^V600E^ tumors transplanted into *Rag2*^−/−^*Il2rg*^−/−^ mice, which lack NK cells, T cells, and B cells versus *Rag1*^−/−^ mice, which lack T and B cells but contain NK cells. Whereas we observed many cDC1 in wild-type (WT) and *Rag1*^−/−^ mice, cDC1 failed to accumulate in tumors transplanted into *Rag2*^−/−^*Il2rg*^−/−^ mice (Figure 2F). In addition, antibody-mediated depletion of NK cells in WT or *Rag1*^−/−^ mice (Figure S2D) resulted in a decrease in cDC1 within *Ptgs1/Ptgs2*^−/−^ BRAF^V600E^ tumors (Figure 2G) irrespective of total tumor mass (Figure S2E). By microscopy, we confirmed reduced cDC1 numbers in tumor sections and further noticed decreased cDC1 clustering within *Ptgs1/Ptgs2*^−/−^ BRAF^V600E^ tumors transplanted into *Rag2*^−/−^*Il2rg*^−/−^ mice or into NK cell-depleted mice (Figures S2F–S2H). Of note, we also observed a reduction in total CD11c^+^MHCII^+^ cells within *Ptgs1/Ptgs2*^−/−^ BRAF^V600E^ tumors in NK cell-depleted mice, suggesting that accumulation of some cDC2 might also depend on NK cells (Figure S2I). NK cell depletion was further associated with a decrease in intratumoral CD8^+^ but not CD4^+^ T cells (Figures S2J and S2K). In contrast to the observed dependence of cDC1 accumulation on intratumoral NK cells, cDC1-deficiency in *Batf3*^−/−^ mice had no impact on NK cell numbers in *Ptgs1/Ptgs2*^−/−^ BRAF^V600E^ tumors (Figure 2H).

Antibody-mediated depletion of NK cells resulted in rapid growth of *Ptgs1/Ptgs2*^−/−^ tumors in WT mice (Figure 2I). This was similar to the loss of immune control seen in *Batf3*^−/−^ mice but NK cell depletion in the latter further exacerbated tumor growth (Figure 2I), arguing that some but not all of the effects of NK cells on tumor control are mediated through cDC1. This might be expected from the fact that NK cells can directly kill tumor cells and produce cytokines with anti-tumor effects (Guillerey et al., 2016). Taken together, these data suggest that NK cells play a key role in anti-tumor immunity in part but not exclusively by promoting intratumoral accumulation and positioning of cDC1.

### NK Cells Are the Major Source of XCL1 and CCL5 in Tumors and Are Directly Inhibited by PGE_2_

**Figure 3:**
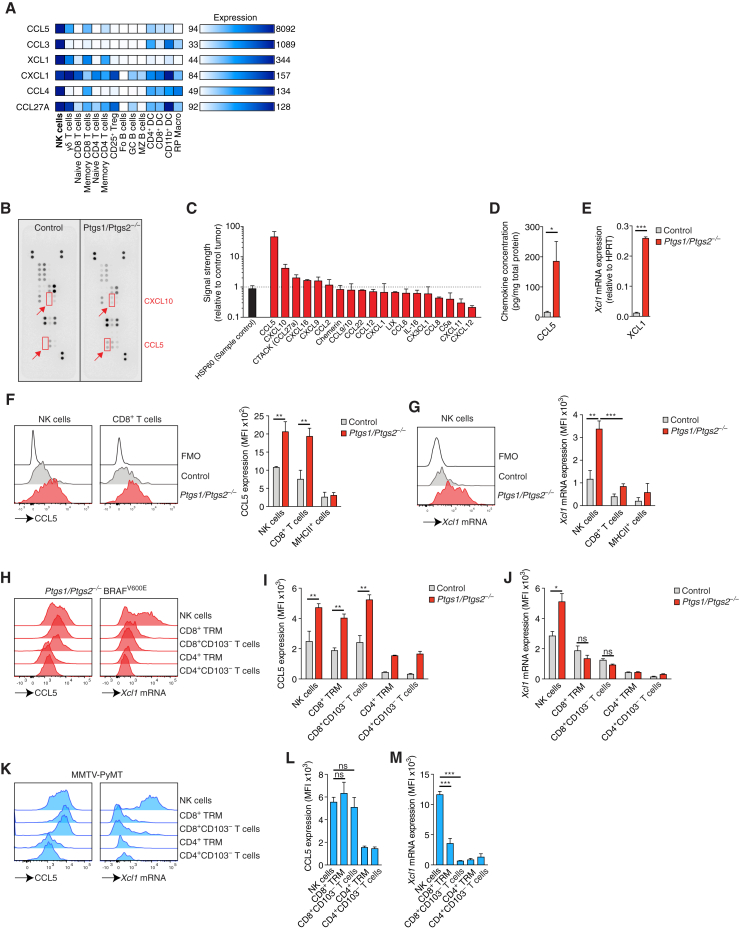
Intratumoral NK Cells Produce CCL5 and XCL1 (A) Selective expression of chemokines by mouse NK cells based on analysis of global gene expression data from splenic immune cells (dataset GSE15907). (B–G) WT mice were injected s.c. with 2 × 10^6^ control or *Ptgs1/Ptgs2*^−/−^ BRAF^V600E^ cells and tumors were analyzed 4 days later. (B) Chemokine expression in tumor lysates determined by protein array. (C) Relative chemokine expression based on densitometric analysis of (B). (D and E) Measurement of (D) CCL5 protein or (E) *Xcl1* mRNA levels in total tumor extracts. (F and G) Flow cytometric analysis of (F) intracellular CCL5 protein or (G) *Xcl1* mRNA in immune cells. FMO, fluorescence minus one. (H–J) As for (B)–(G) but tumors were analyzed 12 days after implantation. (H) Intracellular CCL5 protein and *Xcl1* mRNA levels in NK cells and T cells from a representative *Ptgs1/Ptgs2*^−/−^ BRAF^V600E^ tumor. (I and J) Quantification of intracellular CCL5 protein (I) or *Xcl1* mRNA (J). (K–M) Analysis of CCL5 and *Xcl1* production by immune cells in mammary tumors from female MMTV-PyMT mice. (K) Representative plots showing intracellular CCL5 protein and *Xcl1* mRNA levels. (L and M) Quantification of intracellular CCL5 (L) and intracellular *Xcl1* mRNA (M). Data in (B) and (C) are representative of three independent experiments, bar graphs in (C) depict mean signal from duplicate capture spots ± SD. Data in (D) and (E) are pooled from at least 2 experiments with 3–5 mice per group. In (F), (G), (I), (J), (L), and (M), data are from one of at least two experiments with 3 mice per group represented as mean of each group ± SEM (D–G, I, J, L, and M): n.s., non-significant, ^∗^p < 0.05, ^∗∗^p < 0.01, ^∗∗∗^p < 0.001. See also Figure S3.

**Figure S3:**
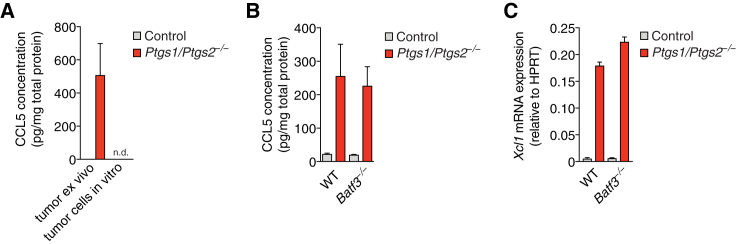
Chemokine Production by Intratumoral NK Cells Does Not Depend on CD103^+^ cDC1, Related to Figure 3 (A) Quantification of CCL5 protein levels in lysates from control or *Ptgs1/Ptgs2*^*−/−*^ tumors 4 days after tumor cell inoculation of WT mice (tumor *ex vivo)* or in lysates from control or *Ptgs1/Ptgs2*^*−/−*^ BRAF^V600E^ cells cultured *in vitro*. (B and C) WT and *Batf3*^*−/−*^ mice were injected s.c. with 2x10^6^ control or *Ptgs1/Ptgs2*^*−/−*^ BRAF^V600E^ cells. 4 days later, tumors were excised, lysed and analyzed for (B) CCL5 protein or (C) *Xcl1* mRNA. Data are representative of two independent experiments with 3-5 tumors per group and shown as mean of each group of mice from one experiment ± SEM n.d., none detected.

Analysis of gene expression data from the Immunological Genome (ImmGen) Project (Heng et al., 2008) indicated that six chemokines, *CCL5*, *CCL3*, *XCL1*, *CXCL1*, *CCL4*, and *CCL27A*, can be expressed by NK cells (Figure 3A). In a protein array, lysates of *Ptgs1/Ptgs2*^−/−^ BRAF^V600E^ early (day 4) tumors displayed 45-fold more CCL5 than COX-sufficient control tumor lysates and a minor (<2-fold) increase in CCL27A (Figures 3B and 3C). Other chemokines putatively produced by NK cells either showed a small reduction between control and *Ptgs1/Ptgs2*^−/−^ BRAF^V600E^ tumors (CXCL1) in the protein array or could not be detected by this analysis (CCL3 and CCL4). We also observed higher CXCL10 levels in *Ptgs1/Ptgs2*^−/−^ BRAF^V600E^ tumors, a chemokine that can be produced by intratumoral DCs to attract T cells (Spranger et al., 2017). High levels of CCL5 protein in lysates from *Ptgs1/Ptgs2*^−/−^ BRAF^V600E^ tumors but not control BRAF^V600E^ tumor lysates were confirmed by cytometric bead array (CBA) analysis (Figure 3D). Of note, we did not find any differences in CCL5 levels between lysates of control or *Ptgs1/Ptgs2*^−/−^ BRAF^V600E^ cells cultured *in vitro*, suggesting that tumor-infiltrating cells rather than the tumor cells themselves are the main source of this chemokine *in vivo* (Figure S3A).

**Figure 4:**
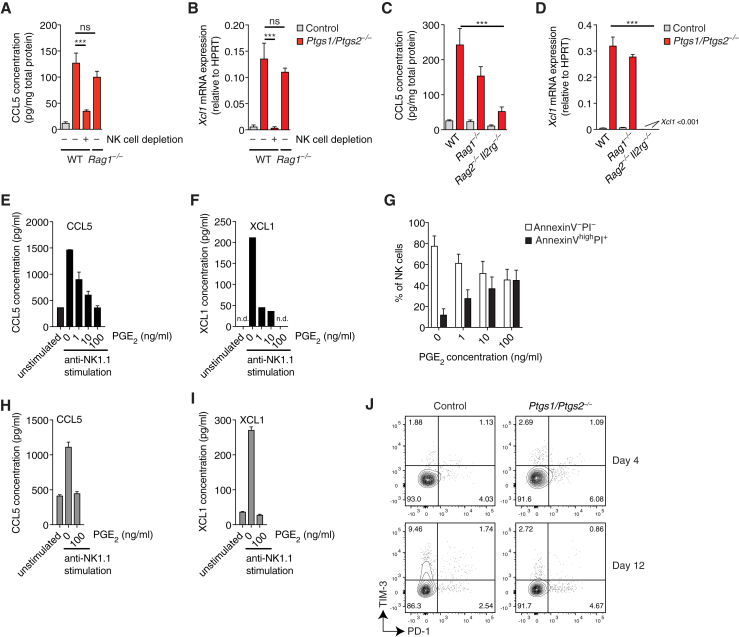
NK Cells Are the Main Source of CCL5 and XCL1 in COX-Deficient Tumors (A and B) WT mice, WT mice depleted of NK cells, or *Rag1*^−/−^ mice were injected s.c. with 2 × 10^6^ control or *Ptgs1/Ptgs2*^−/−^ BRAF^V600E^ cells and analyzed 4 days later for CCL5 protein (A) and *Xcl1* mRNA (B). (C and D) WT mice, *Rag1*^−/−^ and *Rag2*^−/−^*Il2rg*^−/−^ mice were injected s.c. with 2 × 10^6^ control or *Ptgs1/Ptgs2*^−/−^ BRAF^V600E^ cells and analyzed 4 days later for CCL5 protein (C) and *Xcl1* mRNA (D). (E–G) Splenic NK cells from WT mice were cultured with IL-2 and stimulated with plate-bound anti-NK1.1 for 16 hr in the presence or absence of the indicated concentrations of PGE_2_. Culture supernatants were analyzed for CCL5 (E) and XCL1 (F) proteins while NK cells were analyzed for survival by flow cytometric analysis with annexin V and propidium iodide (G). (H and I) NK cells isolated from *Ptgs1/Ptgs2*^−/−^ BRAF^V600E^ tumors were stimulated with plate-bound anti-NK1.1 for 16 hr *in vitro* in the presence or absence of the indicated concentration of PGE_2_. Culture supernatants were analyzed for CCL5 (H) and XCL1 (I) proteins. (J) Expression of TIM-3 and PD-1 on NK cells in BRAF^V600E^ tumors at day 4 and day 12 after tumor transplantation. Data in (A)–(D) are pooled from at least two independent experiments with 3–5 mice per group and represented as mean per group ± SEM. Data from one out of at least two experiments is shown in (E)–(J). Error bars indicate mean of duplicate wells ± SD; (F): n.d., not detected. (A–D): n.s., non-significant, ^∗∗∗^p < 0.001. See also Figure S4.

Because XCL1, the ligand for the chemokine receptor XCR1 expressed by cDC1, was not represented in the protein or the cytometric bead arrays, we analyzed tumor extracts for *Xcl1* mRNA. Similar to CCL5 protein, *Ptgs1/Ptgs2*^−/−^ BRAF^V600E^ but not control BRAF^V600E^ day 4 tumors contained abundant XCL1 transcripts (Figure 3E). By intracellular flow cytometry, NK cells but not MHCII^+^ cells stained positive for CCL5 protein and for *Xcl1* mRNA in *Ptgs1/Ptgs2*^−/−^ BRAF^V600E^ but not control tumors (Figure 3F). Rare infiltrating CD8^+^ T cells at this time point also produced CCL5 but expressed only low levels of *Xcl1* mRNA (Figure 3G). At a later time point (12 days), when T cell infiltration is much more prominent, CCL5 protein was detectable in NK and CD8^+^ T cells in *Ptgs1/Ptgs2*^−/−^ BRAF^V600E^ tumors but *Xcl1* mRNA still remained largely restricted to NK cells and was not highly expressed by any T cells, including TRM (Figures 3H–3J). Similar observations were made in spontaneously developing mammary tumors in MMTV-PyMT mice (Figures 3K–3M). In line with these data, the levels of CCL5 protein and *Xcl1* mRNA in day 4 *Ptgs1/Ptgs2*^−/−^ BRAF^V600E^ tumors were identical between WT and *Batf3*^−/−^ mice (Figures S3B and S3C), but were severely reduced in WT mice depleted of NK cells (Figures 4A and 4B). Similarly, we detected very low levels of CCL5 protein and *Xcl1* mRNA in *Ptgs1/Ptgs2*^−/−^ BRAF^V600E^ tumors transplanted into *Rag2*^−/−^*Il2rg*^−/−^ mice (Figures 4C and 4D) but observed only a minimal, non-significant, reduction of CCL5 and XCL1 expression in *Rag1*^−/−^ mice (Figures 4A and 4B). Therefore, in the absence of PGE_2_, intratumoral NK cells are a major source of XCL1 and CCL5.

**Figure S4:**
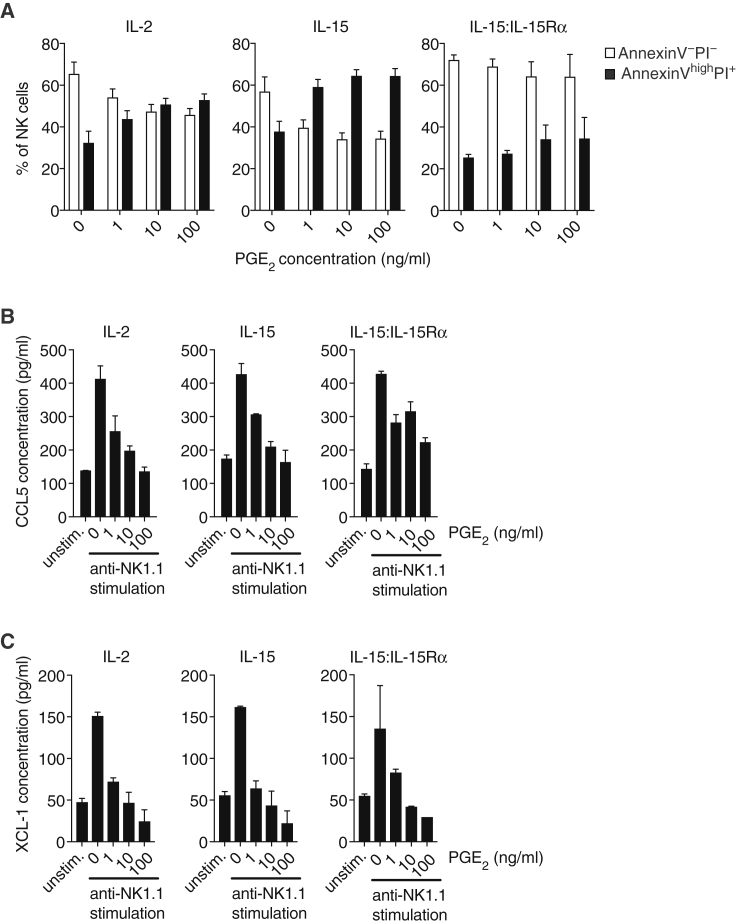
Effect of IL-15 and IL-15:IL-15Rα on PGE_2_-Mediated Inhibition of NK Cell Function, Related to Figure 4 Splenic NK cells from WT mice were cultured for 16h with IL-2, IL-15 or IL-15:IL-15Rα complexes with our without anti-NK1.1 stimulation and in the presence or absence of the indicated concentrations of PGE_2_. (A) Analysis of NK cell survival by flow cytometric analysis with annexin V and propidium iodide. (B and C) Analysis of CCL5 (B) or XCL1 (C) accumulation in culture supernatants. Data shown in A are pooled from three independent experiments and represented as mean of each group across three experiments ± SEM. Data from one of two experiments are shown in B and C as mean of duplicate wells per group ± SD.

Finally, to examine whether PGE_2_ directly impacts NK cell production of CCL5 and XCL1, we isolated splenic NK cells from WT mice and stimulated them through NK1.1, an activatory receptor. Anti-NK1.1 induced secretion of CCL5 and XCL1, which was strongly reduced in a dose-dependent manner in presence of PGE_2_ (Figures 4E and 4F). Survival of NK cells was also markedly reduced by PGE_2_ even in the presence of IL-2 (Figure 4G). IL-15 or IL-15:15Rα complex was able to rescue NK cell survival but not the impaired CCL5 and XCL1 production (Figures S4A–S4C). Similar to splenic NK cells, NK cells isolated from tumors were susceptible to PGE_2_ inhibition (Figures 4H and 4I). Despite its marked effects on function and survival, PGE_2_ did not induce the expression of the co-inhibitory receptors TIM-3 and PD-1 by NK cells *in vitro* (data not shown) or *in vivo* (Figure 4J). These data indicate that NK cells are a direct target of tumor-derived PGE_2_, which decreases cell viability and inhibits production of putative cDC1 chemoattractants.

### XCL1 and CCL5 Mediate Recruitment of cDC1 into Tumors to Promote Immune Control

**Figure 5:**
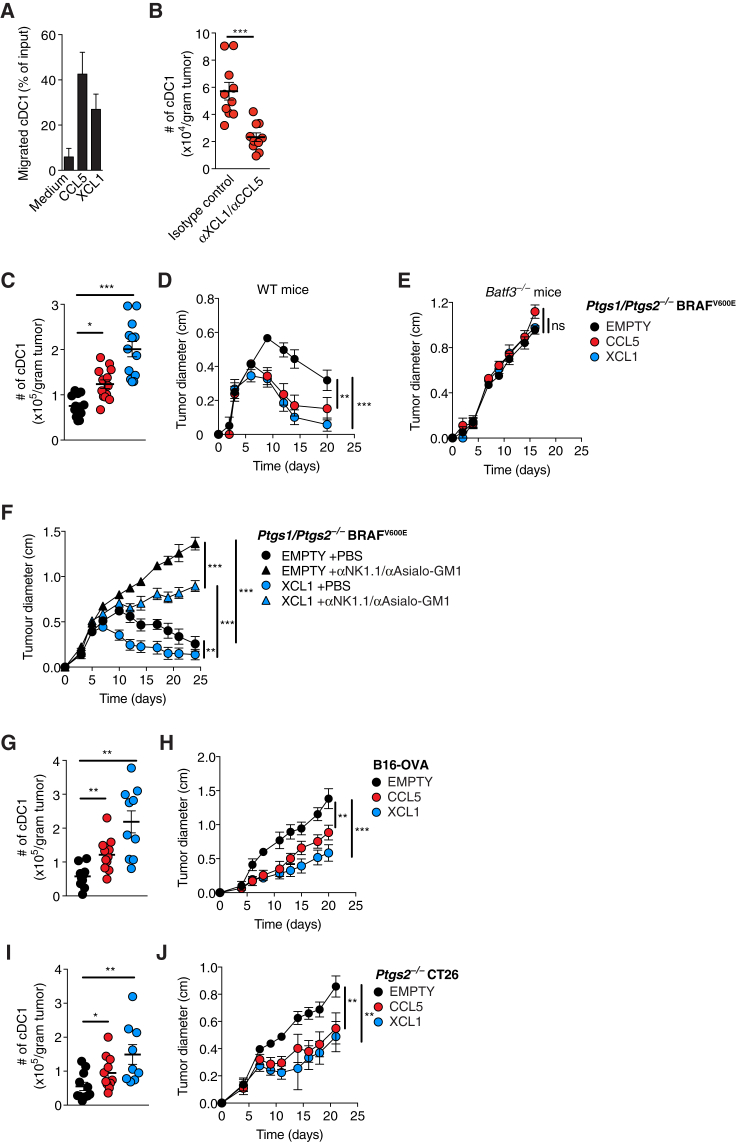
Recruitment of cDC1 into Tumors by XCL1 and CCL5 Promotes Tumor Immune Control (A) Migration of cDC1 toward CCL5 or XCL1. (B) cDC1 accumulation in *Ptgs1/Ptgs2*^−/−^ BRAF^V600E^ tumors in WT mice injected with anti-CCL5 and anti-XCL1 antibodies or the respective isotype-matched controls. (C) Quantification of intratumoral cDC1 4 days after s.c. injection of 2 × 10^6^*Ptgs1/Ptgs2*^−/−^ BRAF^V600E^ cells expressing CCL5 or XCL1 or transduced with an empty vector (EMPTY). (D and E) Growth of the tumors in (C) after s.c. transplantation of 2 × 10^5^ cells into (D) WT or (E) *Batf3*^−/−^ mice. (F) Growth of 2 × 10^5^ EMPTY or XCL1-expressing *Ptgs1/Ptgs2*^−/−^ BRAF^V600E^ cells in WT mice with or without NK cell depletion after s.c. transplantation. (G) Quantification of intratumoral cDC1 4 days after s.c. injection of 2 × 10^6^ B16-OVA cells EMPTY or overexpressing CCL5 or XCL1 into WT mice. (H) Tumor growth following s.c. injection of 2 × 10^5^ B16-OVA cells EMPTY or overexpressing CCL5 or XCL1. (I and J) Same as (G) and (H) but using *Ptgs2*^−/−^ CT26 colorectal cancer cells. Data in (A) are from one of three independent experiments and are shown as mean of duplicate transwells ±SD. Pooled data from at least two experiments are shown in (B)–(J) and represented as mean of each group of mice ± SEM; (B–J): n.s., non-significant, ^∗^p < 0.05, ^∗∗^p < 0.01, ^∗∗∗^p < 0.001. See also Figure S5.

cDC1 express XCR1, the only receptor for XCL1 (Dorner et al., 2009), and CCR1 and CCR5, both of which bind CCL5 (McColl, 2002). Consistent with that expression pattern, cDC1 generated from bone marrow cells *in vitro* migrated efficiently toward CCL5 and XCL1 in a transwell assay (Figure 5A). To investigate whether the two chemokines are necessary for cDC1 recruitment into tumors, we treated WT mice with neutralizing antibodies against CCL5 (αCCL5) and XCL1 (αXCL1) or isotype-matched control antibodies and implanted them with *Ptgs1/Ptgs2*^−/−^ BRAF^V600E^ tumor cells. We found that *in vivo* blockade of CCL5 and XCL1 resulted in markedly reduced cDC1 accumulation within tumors (Figure 5B). These data indicate that cDC1 accumulation in the TME requires CCL5 and XCL1.

Single loss of either CCR5 or XCR1 was not sufficient to block intratumoral cDC1 accumulation (data not shown), likely because of receptor redundancy, and we were not able to test mice doubly deficient in the two receptors because of genetic linkage of the loci. Instead, we used gain-of-function experiments to determine whether CCL5 or XCL1 are sufficient to mediate cDC1 recruitment into the TME. Four days after inoculation of WT mice, cDC1 accumulation was significantly increased in *Ptgs1/Ptgs2*^−/−^ BRAF^V600E^ tumors retrovirally transduced to express CCL5 or XCL1 compared to tumors formed by mock-transduced (EMPTY) cells (Figure 5C). Consistent with increased cDC1 accumulation, *Ptgs1/Ptgs2*^−/−^ BRAF^V600E^ tumors expressing CCL5 or XCL1 showed accelerated rejection in WT mice compared to mock-transduced cells (Figure 5D) but all grew at a comparable rate in *Batf3*^−/−^ mice (Figure 5E). The latter indicates that CCL5- or XCL1-expressing tumors are not intrinsically compromised in their ability to grow *in vivo* but are controlled by the immune system in a cDC1-dependent manner. In NK cell-depleted mice, *Ptgs1/Ptgs2*^−/−^ BRAF^V600E^ tumors expressing XCL1 grew more slowly than mock-transduced cells (Figure 5F), suggesting that XCL1-mediated recruitment of cDC1 can partially compensate for the loss of tumor immune control caused by NK cell ablation.

Next, we extended this analysis to other tumor models. Similar to *Ptgs1/Ptgs2*^−/−^ BRAF^V600E^ tumors, B16-OVA tumors (that do not produce PGE_2_) (Zelenay et al., 2015) expressing CCL5 and XCL1 showed increased accumulation of cDC1 within the TME (Figure 5G) and decreased tumor growth (Figure 5H). A similar trend was observed for tumors formed by *Ptgs2*^−/−^ CT26 colorectal cancer cells in BALB/c mice (Figures 5I and 5J). We conclude that cDC1 accumulation within the TME can be induced by the cDC1-recruiting chemokines CCL5 and XCL1 to improve tumor immune control.

### PGE_2_ Inhibits the Responsiveness of cDC1 to Chemokines

**Figure S5:**
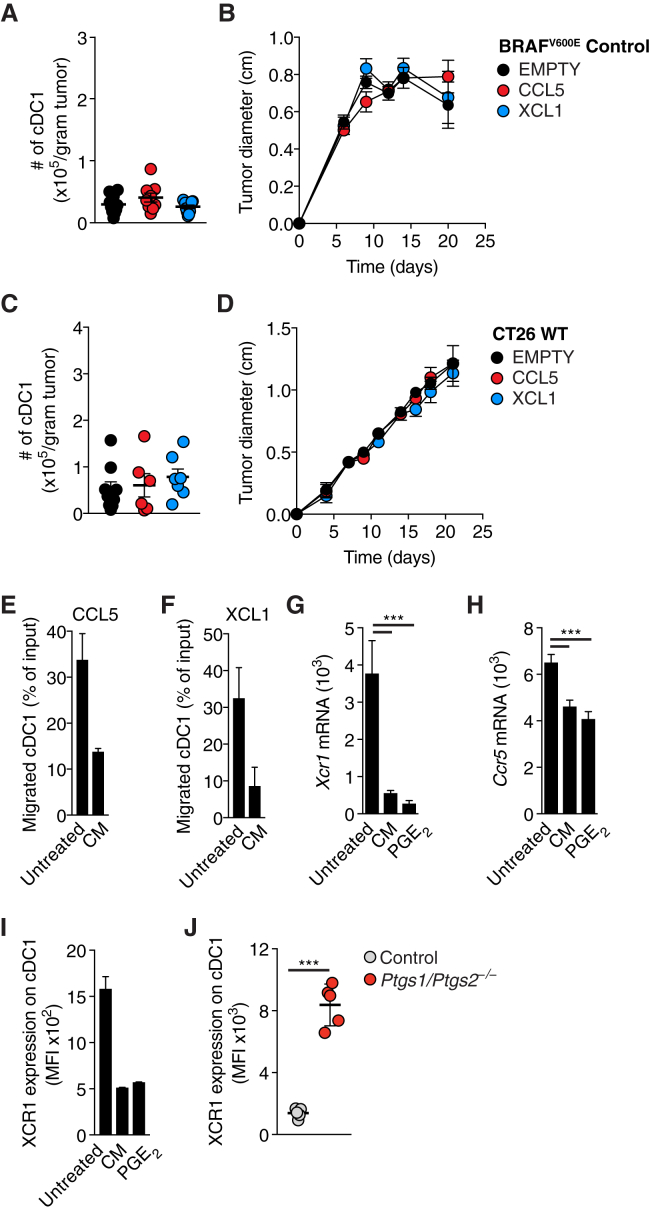
cDC1 Function Is Inhibited by PGE_2_, Related to Figure 5 (A) Quantification of intratumoral cDC1 4 days after s.c. transplantation of 2x10^6^ COX-sufficient control BRAF^V600E^ melanoma cells EMPTY or expressing CCL5 or XCL1. (B) Tumor growth of COX-sufficient control BRAF^V600E^ melanoma cells EMPTY or expressing CCL5 or XCL1 after s.c. transplantation of 2x10^5^ cells into WT mice. (C and D) Same as (A-B) but using CT26 cancer cells. (E–I) CD103^+^ cDC1 from *in vitro* DC cultures were sorted by FACS and incubated for 16h with PGE_2_ (100ng/ml) or conditioned medium (CM) from COX-sufficient control BRAF^V600E^ melanoma cells before testing *in vitro* migration toward (E) recombinant CCL5 or (F) recombinant XCL1. (G-H) Analysis of *Xcr1 (G)* or *Ccr5* (H) mRNA by RT-PCR or XCR1 surface protein by flow cytometry after the incubation period . (J) Flow cytometric analysis of XCR1 surface expression on intratumoral cDC1 isolated from control or *Ptgs1/Ptgs2*^*−/−*^ BRAF^V600E^ tumors. Pooled data from at least two independent experiments are shown in A-D and G, H and depicted as mean of all mice per group ± SEM. Representative data from one of at least three independent experiments are shown in E, F, I and J and depicted as mean per duplicate wells or mice per group from one experiment ± SD.

We further investigated whether CCL5 or XCL1 expression also induced cDC1 accumulation in COX-competent tumors, bypassing PGE_2_-mediated suppression of NK cells. Interestingly, neither CCL5 nor XCL1 expression was able to rescue the low abundance of cDC1 in BRAF^V600E^ tumors (Figure S5A), which all grew similarly to mock-transduced cells in WT mice (Figure S5B). Similar observations were made with COX-sufficient CT26 tumors expressing CCL5 or XCL1 (Figures S5C and S5D). Therefore, chemokine expression alone is not sufficient to recruit cDC1 into PGE_2_-producing tumors, suggesting that PGE_2_ not only suppresses CCL5 and XCL1 production by NK cells but also impairs cDC1 responsiveness to the chemokines. Consistent with that notion, cDC1 exposed to conditioned medium (CM) from PGE_2_-producing tumors were impaired in their migration toward CCL5 and XCL1 (Figures S5E and S5F). Furthermore, cDC1 incubated with CM from PGE_2_-producing cells or with synthetic PGE_2_ downregulated *Xcr1* and *Ccr5* mRNA and XCR1 protein (Figures S5G–S5I). XCR1 downregulation was also seen in cDC1 isolated from BRAF^V600E^ tumors (Figure S5J). These data indicate that PGE_2_ can block the ability of cDC1 to migrate toward the chemokines CCL5 and XCL1 in part by inducing downregulation of the respective receptors.

### Analysis of Human Cancer Datasets Reveals a Close Correlation between NK Cells, Chemokines, and cDC1

**Figure 6:**
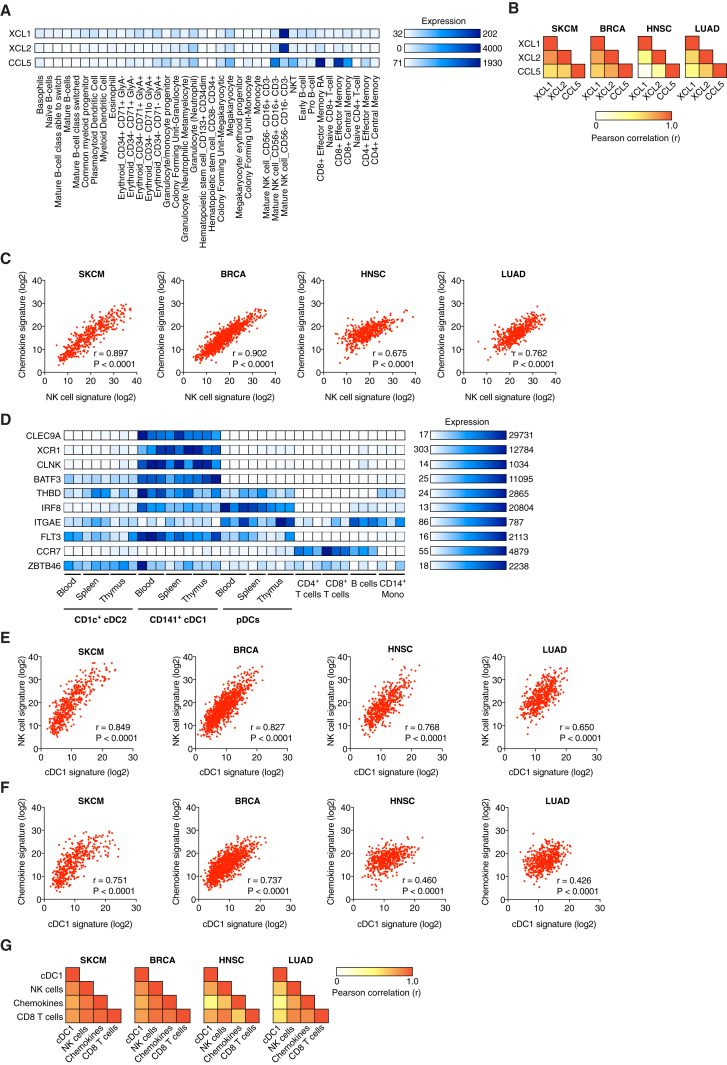
Cross Correlation of Gene Signatures for NK Cells, cDC1, CCL5, XCL1, and XCL2 in Human Cancer (A) Analysis of *CCL5*, *XCL1*, and *XCL2* expression in 38 human hematopoietic cell populations based on global gene expression data (dataset from GSE24759). (B) Heatmap showing Pearson correlation values calculated pairwise between *XCL1*, *XCL2*, and *CCL5* transcript levels in human TCGA datasets for skin cutaneous melanoma (SKCM, n = 460), breast invasive carcinoma (BRCA, n = 1092), head and neck squamous cell carcinoma (HNSC, n = 518), and lung adenocarcinoma (LUAD, n = 506). (C) Correlation between signatures for chemokines and NK cells within TCGA datasets. (D) Identification of cDC1-specific genes in human DC subsets based on global gene expression data (dataset from GSE77671). (E) Correlation of gene signatures specific for cDC1 and NK cells in TCGA datasets. (F) Correlation of gene signatures for chemokines and cDC1 in TCGA datasets. (G) Heatmap showing the Pearson correlation coefficient for the indicated gene signatures in TCGA datasets. r, Pearson correlation coefficient (r); p, p value. See also Figure S6.

To determine whether NK cells can similarly serve as a source of XCL1 and CCL5 in humans, we analyzed a gene expression dataset of 38 populations of human hematopoietic cells (Novershtern et al., 2011). We additionally probed for XCL2, a paralog of XCL1 that is found in humans but not mice and also binds to XCR1 with high affinity (Fox et al., 2015). Both *XCL1* and *XCL2* were highly expressed within the CD56^−^ (often referred to as CD56^dim^) subset of NK cells (Figure 6A). *CCL5* was expressed in CD56^−^ and CD56^+^ NK cells, as well as in other immune cell populations such as effector and memory CD8^+^ T cells (Figure 6A). Therefore, human NK cells can produce the chemokines CCL5, XCL1, and XCL2 and are a rare source of the latter two, at least in blood under steady-state conditions.

**Figure S6:**
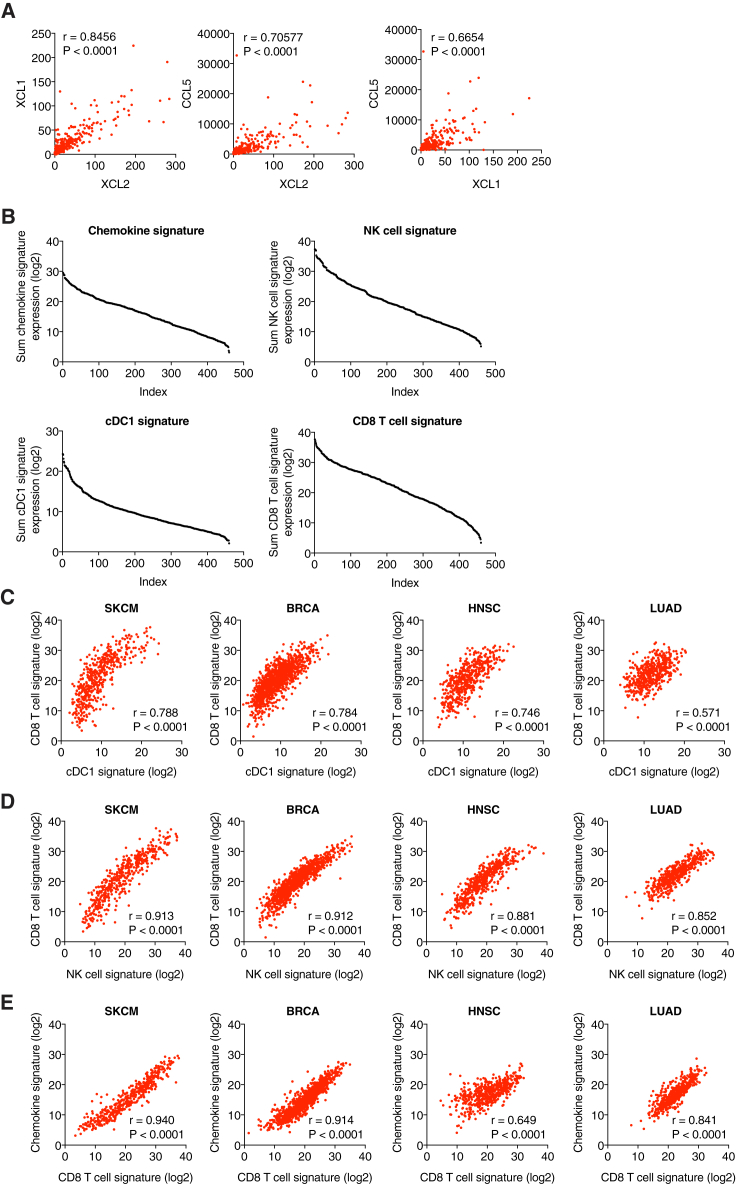
Expression of Gene Signatures for cDC1, NK cells, CD8 T Cells, and Chemokines in Human Cancer Patients, Related to Figure 6 (A) Scatterplots showing the correlation of transcript levels for *XCL1* versus *XCL2*, *CCL5* versus *XCL2* and *CCL5* versus *XCL1* for all patients from the TCGA SKCM dataset. (B) Distribution of the sum expression of indicated signature genes for all patients from the TCGA datasets SKCM, ordered from high to low. (C–E) Scatterplots showing the correlation between gene signatures within patient cohorts from TCGA datasets. (C) Signatures for CD8 T cells and cDC1. (D) Signatures for CD8 T cells and NK cells. (E) Signatures for chemokines and CD8 T cells. Pearson correlation coefficient (r) and P value are shown throughout.

To investigate the presence of these chemokines in human cancers, we looked at tumor gene expression data from The Cancer Genome Atlas (TCGA). When analyzing TCGA datasets for skin cutaneous melanoma (SKCM, n = 470 patients), breast invasive carcinoma (BRCA, n = 1098), head and neck squamous cell carcinoma (HNSC, n = 528), and lung adenocarcinoma (LUAD, n = 585), we observed a positive correlation between the three chemokines *XCL1*, *XCL2*, and *CCL5* that was highly significant in each dataset (Figures 6B and S6A), consistent with the notion that they might all be produced by the same intratumoral cell type. To assess whether that type could be NK cells, we compared a chemokine gene expression signature containing *XCL1*, *XCL2*, and *CCL5* with a broad signature of NK cells (Figure S6B). We observed a highly significant, positive correlation between the two gene signatures in all four TCGA datasets (Figure 6C).

Next, we investigated whether cDC1 accumulation in human tumors correlated with the abundance of NK cells and the chemokines. To identify transcripts best suited to identify cDC1, we utilized a recently published gene expression dataset for DC populations in different human tissues (Heidkamp et al., 2016). We derived a human cDC1 signature that included *CLEC9A* (Poulin et al., 2012), *XCR1* (Bachem et al., 2010, Dorner et al., 2009, Fox et al., 2015), *CLNK*, and the transcription factor *BATF3* (Robbins et al., 2008) (although the latter was also expressed at low levels in cDC2). We excluded *CCR7*, *THBD* (CD141/BDCA3), *IRF8*, *ITGAE* (*CD103*), *FLT3*, and *ZBTB46*, which have previously been used as cDC1 markers (Broz et al., 2014, Roberts et al., 2016) but, in our analysis, displayed promiscuous expression (Figure 6D). In all cancer types, our cDC1 signature showed a high degree of positive correlation with both the gene signatures for NK cells (Figure 6E) and the chemokine signature (Figure 6F). The 3-way correlation always reached significance but was most profound in melanoma and breast cancer (Figure 6G). A CD8 T cell signature (Figure S6B) also correlated with the signatures for cDC1, NK cells, and chemokines (Figures 6G and S6C–S6E), consistent with the notion that the interplay between NK cells and cDC1 favors CD8^+^ T cell-dependent anti-tumor immunity.

### Gene Signatures of NK Cells and cDC1 Positively Correlate with Cancer Patient Survival

**Figure 7:**
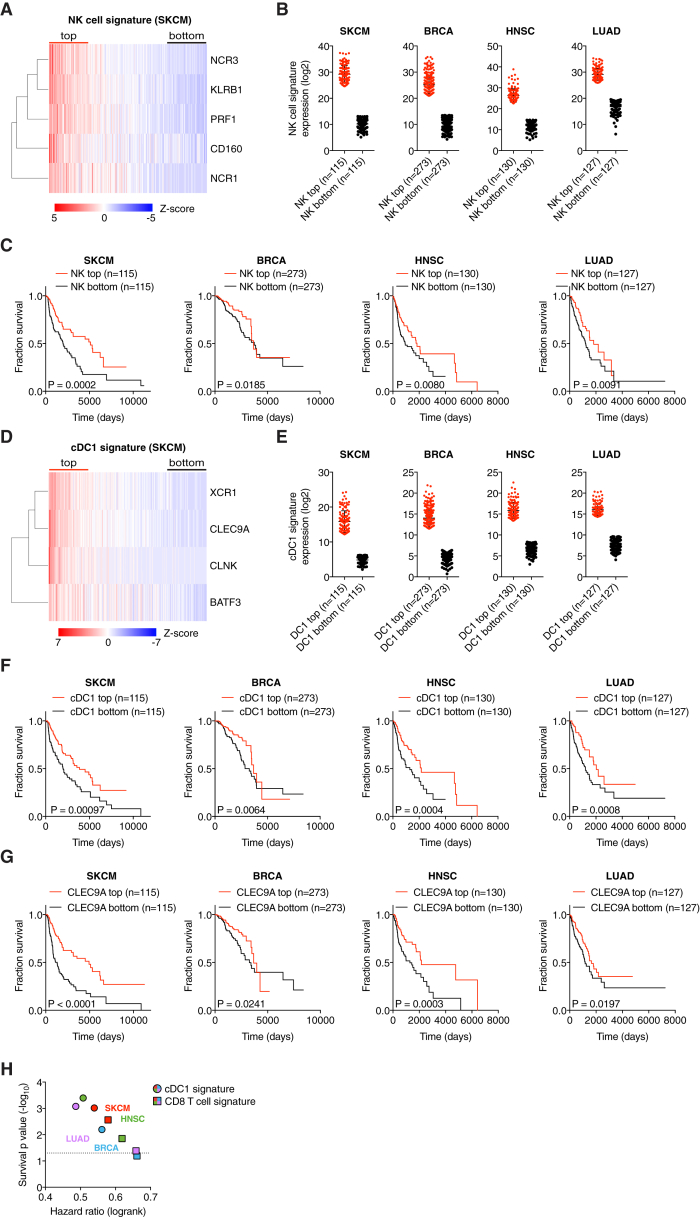
Gene Signatures of NK Cells and cDC1 Positively Correlate with Cancer Patient Survival (A) Heatmap showing the ordered, z-transformed expression values for NK cell-specific genes in melanoma patients. (B) Expression of NK cell signature genes for top and bottom quartiles of TCGA datasets. (C) Prognostic value of the NK cell signature for overall survival of human cancer patients comparing top and bottom quartiles. (D) Heatmap showing the ordered, z-transformed expression values for cDC1-specific genes in melanoma patients. (E) Expression of cDC1 signature genes for top and bottom quartiles of indicated TCGA datasets. (F and G) Prognostic value of the cDC1 gene signature (F) or of *CLEC9A* expression levels (G) for cancer patient overall survival comparing top and bottom quartiles. (H) Hazard ration comparison of the cDC1 and a CD8 T cell signature as an indicator of overall survival. The dotted line indicates a p value of 0.05. Data in (B) and (E) are represented as mean ± SD. p, p value; n, number of data points in the analysis. See also Figure S7 and Table S1.

Finally, we assessed the degree to which these observations related to disease outcome. Notably, higher expression of NK signature genes in tumor samples (Figures 7A and 7B) was significantly associated with patient survival in all cancer types (Figure 7C; Table S1). Similarly, stratification of patients by expression of cDC1-associated genes (Figures 7D and 7E) indicated that a higher cDC1 signature in tumors is positively associated with survival (Figure 7F; Table S1). Of note, a single cDC1 marker, *CLEC9A*, was also prognostic of patient survival (Figure 7G) and the cDC1 signature was at least as powerful a predictor of cancer patient survival as a CD8 T cell signature (Figure 7H).

**Figure S7:**
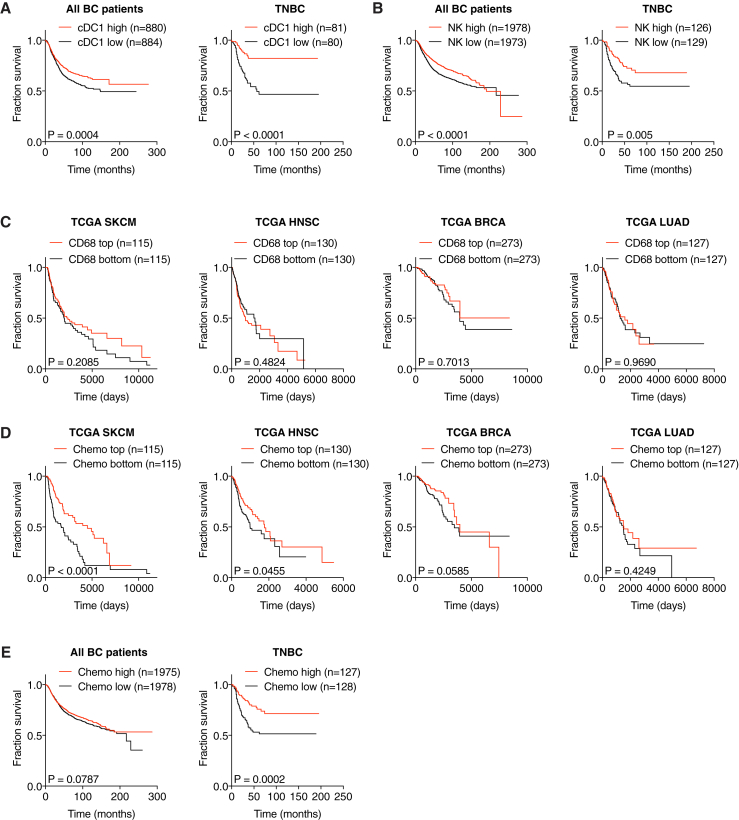
Prognostic Value of Chemokine Expression for Overall Survival, Related to Figure 7 (A and B) Survival analyses of a human breast cancer patient cohort with associated gene expression data available at the KM plotter site (http://kmplot.com). All breast cancer patients from the dataset or a sub-group of patients diagnosed with triple-negative breast cancer (TNBC) were split into the top and bottom half for expression of signature genes and compared for overall survival. (A) Prognostic value of a cDC1 signature (*CLNK*, *BATF3*, *XCR1*) for all breast cancer patients (n = 1764) or TNBC patients (n = 161). Please note that *CLEC9A* transcript is absent in this particular dataset and cannot be included in the signature. (B) Prognostic value of a NK cell signature (*NCR1*, *NCR3*, *KLRB1*, *CD160*, *PRF1*) for all breast cancer patients (n = 3951) or patients with TNBC (n = 255). (C) Prognostic value of CD68 expression levels in tumor biopsies for overall survival of human cancer patients from TCGA datasets. (D and E) Prognostic value of a chemokine signature (*XCL1*, *XCL2* and *CCL5*) for overall survival of human cancer patients from (D) TCGA datasets as indicated or (E) human breast cancer patients available at the KM plotter site. p = p value, n = number of data points in the analysis.

Consistent with the analyses in TCGA datasets, we found a similar positive correlation of NK cell and cDC1 gene signatures in an independent cohort of breast cancer patients (Figures S7A and S7B) in which cancers were further classified into clinical groups, including the triple-negative breast cancer subtype (TNBC) that has poor prognosis. Strikingly, TNBC samples showed a very significant positive association between cDC1 and NK cell signature genes and survival (Figures S7A and S7B), which was even more pronounced than when all breast cancer patients were analyzed without separating by tumor subgroup. Altogether, these data indicate across a wide array of human tumors that those with the highest NK cell and cDC1 content display the best prognosis. Importantly, high NK cell and cDC1 content was not merely a proxy for cancers with high overall immune infiltration as expression of monocyte/macrophage-specific genes such as *CD68* or *CD14* in tumor samples was not positively correlated with patient survival in any type of cancer (Figure S7C and data not shown). Thus, our analysis reveals the importance of the quality rather than the quantity of the immune infiltrate and the favorable consequences of NK cell and cDC1 recruitment.

Similar to the analyses of cDC1 and NK cell gene signatures, we observed a positive correlation with survival when we ranked melanoma, HNSC and TNBC patients according to expression of *CCL5*, *XCL1*, and *XCL2* (Figures S7D and S7E). However, in lung and breast cancer, with the exception of TNBC, the association between higher chemokine signature and survival did not reach significance (Figure S7D), despite them all displaying an association of survival with cDC1 signatures. This discrepancy is likely due to chemokine redundancy and additional mechanisms contributing to the accumulation of cDC1 within some tumors. Finally, it is possible that pleiotropic chemokines such as CCL5 can contribute to tumor progression by recruiting regulatory T cells (Velasco-Velázquez et al., 2014), obscuring the prognostic value of the three chemokine signature (data not shown).

## Discussion

Myeloid cells such as macrophages and DCs within the TME shape tumor development and anti-cancer immunity. cDC1 fulfill a unique role in promoting the latter through their ability to transport tumor antigens to tumor draining LNs for T cell priming, to produce chemokines that recruit T cells into the TME, and to locally secrete IL-12 and restimulate tumor-infiltrating T cells. Because of these functions, the abundance of intratumoral cDC1s in tumors has previously been suggested to correlate with immune-mediated control and favorable outcome in both mice and humans (Broz et al., 2014, Salmon et al., 2016, Spranger et al., 2015, Spranger et al., 2017). However, the mechanisms that control the accumulation of cDC1 within tumors remain poorly understood. Here, we uncover a key role for NK cells in the production of chemoattractants, including CCL5 and XCL1/2, that are necessary for the accumulation of cDC1 in incipient tumors and for tumor immune control. We also show that this axis can be subverted by tumor-derived PGE_2_, which both impairs NK cell function and causes downregulation of the XCR1 and CCR5 chemokine receptors on cDC1. Finally, we suggest that intratumoral cDC1 accumulation in human tumors is regulated by a similar mechanism as in mice and is positively correlated with favorable patient outcome in several different types of cancer. These findings illuminate the early events leading to tumor immunity and uncover a new checkpoint in immunity that could form the basis for novel cancer immunotherapies and for improving current ones.

NK cells contribute to anti-tumor immunity in mice and are associated with good prognosis in human cancer patients (Palucka and Coussens, 2016, Vesely et al., 2011). They can secrete cytostatic cytokines such as interferon gamma (IFN-γ) and can directly kill tumor cells, functions that render NK cells an attractive potential target for immunotherapy. Our analyses indicate an additional, so far unrecognized, function of NK cells in the recruitment of cDC1 into the TME. Interestingly, exclusion of cDC1 in lung adenocarcinoma has been reported to coincide with a lack of intratumoral NK cells (Lavin et al., 2017), independently supporting the idea that NK cells contribute to the accumulation of cDC1 in tumors. At the same time, our data raise the question of how NK cells recognize tumor cells and accumulate within incipient tumors. Cells undergoing neoplastic transformation often show increased expression of ligands for NK cell activating receptors (Diefenbach et al., 2001, Gasser et al., 2005), which endow NK cells with the ability to participate in tissue stress surveillance responses (Hayday, 2009, Raulet and Guerra, 2009). Therefore, activation of NK cell receptors by ligands expressed by transformed cells might be key for innate anti-tumor immunity and, as revealed here, also sets in motion adaptive immunity through production of cDC1 chemoattractants. Consistent with this notion, stimulation of the activating receptor NK1.1 on NK cells *in vitro* resulted in secretion of both CCL5 and XCL1. However, the degree to which activating ligands expressed by cancer cells versus additional signals (e.g., from cytokines such as type I IFNs or IL-15) (Delconte et al., 2016) contribute to NK cell-dependent ignition of anti-cancer immunity remains to be elucidated.

Although fully differentiated cDC1 can be detected in blood, especially in humans, cDC1 that accumulate in tissues in steady-state are thought to develop from committed precursors (pre-cDC1) that originate in bone marrow and travel via blood to seed peripheral organs (Grajales-Reyes et al., 2015, Liu et al., 2009, Schlitzer et al., 2015). However, it is not known whether “emergency” needs for cDC1, such during tumor development, are met by increased pre-cDC1 recruitment. Notably, pre-cDC1 do not express the chemokine receptors XCR1 or CCR5 (Grajales-Reyes et al., 2015), implying that they cannot respond to CCL5 and XCL1 secreted by intratumoral NK cells. Similarly, the steady-state tissue colonization of tissues by DCs is NK cell-independent. Therefore, while it will be important to investigate the degree to which pre-cDC1 might be selectively recruited to tumors, our findings, including from *ex vivo* migration assays, suggest that the NK cell-dependent cDC1 accumulation could additionally rely on the recruitment and/or retention of differentiated cDC1, either from the circulation or surrounding tissue.

In murine tumors, intratumoral cDC1 were located in very close proximity to NK cells and both cell types were often situated in multicellular clusters within the TME. It seems likely that these clusters are a result of the chemotactic cues provided by NK cells, but might additionally be supported by chemokines secreted by cDC1 (e.g., CXCL9 and CXCL10) that could attract NK cells via CXCR3 (Spranger et al., 2017, Wendel et al., 2008). The observed clustering of cDC1 and NK cells within the TME might ensure further communication between these two cell types, eventually leading to mutual activation of cDC1 and NK cells and facilitating anti-tumor immunity, similar to reciprocal activation of cDC1 and XCL1-secreting T cells in lymph nodes and within the intestine (Brewitz et al., 2017, Ohta et al., 2016). Consistent with this possibility, a very recent study suggests that cDC1-derived IL-12 is essential for the anti-tumor activity of NK cells (Mittal et al., 2017) while NK cell-derived IFN-γ sustains production of IL-12 by cDC1 (Alexandre et al., 2016). Nevertheless, it is possible that, in some instances, infiltrating T or other cells producing CCL5, XCL1, and/or XCL2 can substitute for NK cells in maintaining the recruitment of cDC1.

The generation of PGE_2_ by elevated COX activity is a mechanism by which tumors can evade anti-tumor immunity (Zelenay et al., 2015). Our results indicate that a key target of PGE_2_ is NK cells, which fail to accumulate in PGE_2_-producing tumors and are further impaired in their survival and ability to produce CCL5 and XCL1. PGE_2_ likely has additional effects on NK cells, such as inducing the downregulation of NK cell activatory receptors or cytotoxic effector molecules (Kalinski, 2012). In addition, autocrine PGE_2_ decreases the expression of activating NK cell receptor-ligands on tumor cells (Pietra et al., 2012). However, in addition to targeting NK cell activity, PGE_2_ also hinders cDC1 directly, by causing downregulation of the chemokine receptors that promote recruitment into tumors. Therefore, PGE_2_ impairs anti-tumor immunity by acting on at least two cellular layers of the innate immune system, NK cells and cDC1. In addition, PGE_2_ is known to also directly suppress cytotoxic T cell action (Chen et al., 2015, Su et al., 2011) underscoring its role as a major immunosuppressive mediator that interferes with multiple aspects of anti-cancer immunity.

In a previous study, the ratio between transcripts from a set of genes enriched in cDC1 and levels of transcripts expressed by other myeloid cells proved to be a prognostic marker for cancer patient survival (Broz et al., 2014). Furthermore, CCR7 transcript levels in a cohort of melanoma patients correlated with survival, pointing to a potential role for CCR7-expressing cells, including cDC1, in anti-tumor immunity (Roberts et al., 2016). However, the transcripts used in those studies are not entirely selective for cDC1 and, therefore, we derived a new cDC1 gene signature based on the transcriptome of DC subsets across several human tissues (Heidkamp et al., 2016). The selectivity of the cDC1 signature described here was independently confirmed in a recent unbiased genomic profiling of human DC subsets and monocytes by single-cell RNA-sequencing, which established cDC1-restricted expression of *CLEC9A*, *XCR1*, *CLNK*, and *BATF3* (Villani et al., 2017). This new cDC1 signature provides a powerful means to demonstrate a positive association of cDC1 with patient survival in several human cancers, including metastatic melanoma, breast cancer, head and neck squamous cell carcinoma, and lung adenocarcinoma. Interestingly, *CLEC9A* as a single cDC1-specific marker showed almost identical prognostic value in these analyses, underscoring the strong discriminatory power of *CLEC9A* in identifying cDC1 in tissues, as recently confirmed (Villani et al., 2017). We therefore propose that *CLEC9A* should be used for assessing cDC1 content in tumors, either as a single marker or in combination with other strictly cDC1-specific genes such as *XCR1*. However, XCR1 expression on intratumoral cDC1 has to be carefully evaluated, given our observation that tumor-derived PGE_2_ can induce its downregulation.

Similar to cDC1 in mice, human cDC1 seem to be very rare in tumors and are often excluded from samples associated with tumor progression (Lavin et al., 2017). In line with this, we found a very low abundance of cDC1-specific transcripts in some TCGA datasets such as colorectal cancer, precluding us to evaluate the role of cDC1 in that cancer (data not shown). Interestingly, key enzymes for PGE_2_ production such as COX-2 are often overexpressed in colorectal cancer and associated with poor prognosis (Dannenberg and Subbaramaiah, 2003), suggesting that PGE_2_ might be one of the mechanism responsible for the scarcity of cDC1 in the TME of some human tumors. In line with this, we detected a significant negative correlation between the transcript levels of *PTGS2* and *XCL1* or *XCL2* in some but not all the TCGA patient datasets analyzed in our study (data not shown). Further studies are necessary to determine the exact contribution of PGE_2_ to cDC1 scarcity in human cancer.

Our findings on the interplay between NK cells and cDC1 within the TME have several therapeutic implications. First, our analyses of human cancer samples clearly establish that dearth of cDC1 is associated with poor prognosis of patient outcome, as previously suggested (Broz et al., 2014). This implies that cDC1 have a role in spontaneous anti-tumor immunity in humans, as in mice. Given that intratumoral cDC1 are also essential for T cell-based therapies in mouse tumor models (Broz et al., 2014, Salmon et al., 2016, Sánchez-Paulete et al., 2016, Spranger et al., 2015), our data further indicate that a low frequency of cDC1 might be one reason for the low response rate of cancer patients to immune checkpoint blockade. It would be of great interest to establish whether cDC1 accumulation (e.g., determined by assessing cDC1-specific transcripts such as *CLEC9A*) can serve as a predictive biomarker for the outcome of such treatments. Second, our data indicate that increasing the accumulation of intratumoral cDC1 enhances tumor immune control even in the absence of innate immune stimuli that deliberately promote cDC1 activation. Locally stimulating intratumoral NK cells or developing XCR1 ligands to attract cDC1 into the TME could be an attractive therapeutic means of eliciting anti-tumor immunity and increasing the response rate to immunotherapy. On this note, it seems intuitive to preferentially target the XCL1/XCL2-XCR1 axis rather than CCL5 to guide cDC1 into tumors, thereby ensuring that CCL5-mediated recruitment of tumor-promoting immune cells such as macrophages or regulatory T cells is avoided. Therapeutic strategies aiming to increase cDC1 numbers in tumors might benefit from combination with COX-inhibitors, especially in tumors that show high levels of PGE_2_ production. Finally, it is likely that additional immunosuppressive mechanisms contribute to excluding NK cells and cDC1 from tumors. Identifying such factors may help develop new strategies to augment cDC1 recruitment into tumors and increase the fraction of patients benefiting from cancer immunotherapy.

## STAR★Methods

### Key Resources Table

**Table:** 

REAGENT or RESOURCE	SOURCE	IDENTIFIER
**Antibodies**		

NK1.1 (PK136)	Biolegend	108712
Asialo-GM1 (Poly21460)	Biolegend	146002
CCL5 (Polyclonal Goat IgG)	R&D Systems	AF478
CCL5 (#53405)	R&D Systems	MAB478
XCL1 (Polyclonal Goat IgG)	R&D Systems	AF486
XCL1 (#80222)	R&D Systems	MAB486
CD3e BV605 (17A2)	Biolegend	100237
CD4 PerCP/C5.5 (RMA4.5)	Biolegend	100540
CD8α FITC (53-6.7)	BD Biosciences	553031
CD8β FITC (53-5.8)	Biolegend	140404
CD11b BV711 (M1/70)	Biolegend	101242
CD11c APC-eF780 (N418)	ebioscience	47-0114-82
CD24 PE (M1/69)	BD Biosciences	553262
CD16/CD32 (2.4G2)	BD Biosciences	553142
CD45.2 AF700 (104)	Biolegend	109822
CD49a APC (HMα1)	Biolegend	142606
CD49b PE (DX5)	BD Biosciences	553858
CD64 PE/Cy7 (X54-5/7.1)	Biolegend	139313
CD103 APC BV786 (M290)	BD Biosciences	564322
DNGR-1 PE (1F6)	C. Reis e Sousa	N/A
CCL5 PE (2E9/CCL5)	Biolegend	149103
MHC class II (MHC II) I-a/I-E FITC (M5/114.15.2)	ebioscience	11-5321-85
NK1.1 PE (PK136)	Biolegend	108708
PD-1 BV785 (29F.1A12)	Biolegend	135225
TCRβ FITC (H57-597)	BD Biosciences	553171
TCRγδ PE (GL3)	Biolegend	118108
TIM-3 PE/Cy7 (RMT3-23)	Biolegend	119716
XCR1 BV421 (ZET)	Biolegend	148216
IRF8 APC (V3GYWCH)	ebioscience	17-9852-80
Granzyme B Pacific Blue (GB11)	Biolegend	515408
CD31 AF647 (clone Mec13.3)	Biolegend	102515
CD103 (Polyclonal goat)	R&D Systems	AF1990
Collagen IV (Polyclonal rabbit)	Abcam	Ab6586

**Bacterial and Virus Strains**		

pMSCV-IRES-mCherry	Addgene	#52114

**Critical Commercial Assays**		

PrimeFlow RNA Assay Kit	Thermo Fischer Scientific	88-18005-210
Proteome Profiler Mouse Chemokine Array Kit	R&D Systems	ARY020
Mouse RANTES (CCL5) Flex Set, Cytometric Bead Array	BD Biosciences	558345
Foxp3/Transcription factor staining buffer set	ebiosciences	00-5523-00
RNeasy Mini Kit	QIAGEN	https://www.qiagen.com/us/shop/sample-technologies/rna/total-rna/rneasy-mini-kit/#orderinginformation

**Deposited Data**		

KM plotter	Szász et al., 2016	http://kmplot.com
The Cancer Genome Atlas (TCGA)	Firehose, The Broad Institute	https://gdac.broadinstitute.org/
GSE15907	Immunological Genome (ImmGen) Project	GSE15907; https://www.ncbi.nlm.nih.gov/gds
GSE24759	Novershtern et al., 2011	GSE24759; https://www.ncbi.nlm.nih.gov/gds
GSE77671	Heidkamp et al., 2016	GSE77671; https://www.ncbi.nlm.nih.gov/gds

**Experimental Models: Cell Lines**		

Control BRAF^V600E^	C. Reis e Sousa (Zelenay et al., 2015)	N/A
*Ptgs1/Ptgs2*^*−/−*^ BRAF^V600E^	C. Reis e Sousa (Zelenay et al., 2015)	N/A
CT26.WT	The Francis Crick Institute	N/A
*Ptgs2*^*−/−*^ CT26	C. Reis e Sousa (Zelenay et al., 2015)	N/A
4T1	The Francis Crick Institute	N/A
*Ptgs1/Ptgs2*^*−/−*^*4T1*	C. Reis e Sousa (Zelenay et al., 2015)	N/A
B16-OVA	The Francis Crick Institute	N/A
GP2-293	C. Reis e Sousa	N/A

**Experimental Models: Organisms/Strains**		

MMTV-PyMT (Tg(MMTV-PyVT)634Mul)	Ilaria Malanchi	N/A
*Batf3*^*−/−*^*(*B6.129S6(C)-Batf3^*tm1Kmm*^*)*	Kenneth M. Murphy	N/A
*Rag1*^*−/−*^*(*B6.129S4-Rag1^*tm1Bal*^)	The Francis Crick Institute	N/A
*Rag2*^*−/−*^*Il2rg*^*−/−*^ (Rag2tm1Fwa Il2rgtm1Wjlx)	The Francis Crick Institute	N/A

**Oligonucleotides**		

Primer XCL1-Forward: GGCCCGGGGATCCATGGATGAGA CTTCTCCTCCT	This paper	N/A
Primer XCL1-Reverse: CGGCCAACCGGCTCGAGTTACCC AGTCAGGGTTA	This paper	N/A
Primer CCL5-Forward: GGCCCGGGGATCCATGGATGAAG ATCTCTGCAG	This paper	N/A
Primer CCL5-Reverse: CGGCCAACCGGCTCGAGCTAGCT CATCTCCAAATA	This paper	N/A
RT-PCR Primer Xcl1-Forward: CTTTCCTGGGAGTCTGCTGC	C. Carvalho-Pinto	N/A
RT-PCR Primer Xcl1-Reverse: CAGCCGCTGGGTTTGTAAGT	C. Carvalho-Pinto	N/A
RT-PCR Primer HPRT-Forward: TCAGTCAACGGGGGACATAAA	S. Sakaguchi	N/A
RT-PCR Primer HPRT-Reverse: GGGGCTGTACTGCT	S. Sakaguchi	N/A

**Recombinant DNA**		

Plasmid: VSV-G	C. Reis e Sousa	N/A

**Software and Algorithms**		

GraphPad Prism v7	GraphPad	https://www.graphpad.com/scientific-software/prism/
Imaris v8.3.1	Bitplane	http://www.bitplane.com
R version 3.4.2	R Project	https://www.r-project.org

### Contact for Reagent and Resource Sharing

Further information and requests for resources and reagents should be directed to the Lead Contact, Caetano Reis e Sousa (caetano@crick.ac.uk). Please note that additional Material Transfer agreements will be necessary to obtain BRAF^V600E^ melanoma cells or *Batf3*^*−/−*^*, Rag2*^*−/−*^*Il2rg*^*−/−*^ and MMTV-PyMT mice.

### Experimental Model and Subject Details

#### Mice

C57BL/6, MMTV-PyMT transgenic C57BL/6 mice, *Batf3*^*−/−*^, *Rag1*^*−/−*^, *Rag2*^*−/−*^*Il2rg*^*−/−*^ and BALB/c mice were bred at The Francis Crick Institute under specific pathogen-free conditions. Mice were used at 6-12 weeks of age, gender-matched and littermates of the same sex were randomly assigned to treatment or control groups in all experiments. All animal experiments were performed in accordance with national and institutional guidelines for animal care and were approved by the Francis Crick Institute Biological Resources Facility Strategic Oversight Committee (incorporating the Animal Welfare and Ethical Review Body) and by the Home Office, UK.

#### Cell lines and primary cell cultures

Mycoplasma negative BRAF^V600E^ melanoma, CT26 colorectal cancer, 4T1 breast cancer, B16-OVA and GP2-293 cell lines were and cultured in complete RPMI medium (RPMI 1640 with 10% fetal calf serum, 50μM 2-mercaptoethanol, 100U/ml Penicillin, 100μg/ml Streptomycin, 292ng/ml L-Glutamin). COX-sufficient control and *Ptgs1/Ptgs2*^*−/−*^ cell lines were generated by CRISPR/Cas9-mediated gene editing as described previously (Zelenay et al., 2015). *In vitro* differentiation of CD103^+^ cDC1 was performed with primary bone marrow cells from female C57BL/6 mice at 6-12 weeks of age using the induced CD103 DC protocol (Mayer et al., 2014). DCs were harvested 12-14 days after the start of the culture and used for experiments.

### Method Details

#### Tumor cell injections

Cells were harvested by trypsinization and washed three times in PBS. 2x10^5^ or 2x10^6^ cells were injected s.c. in 100μl endotoxin-free PBS on the flank of recipient mice. Tumor growth was measured using a digital caliper. Tumor diameters stated in the figures refer to the average of the longest diameter and its perpendicular for each tumor.

#### NK cell depletion *in vivo*

For depletion of NK cells, mice were injected i.p. with 100μl of an antibody cocktail containing anti-NK1.1 (clone PK136, 40μg/mouse) and anti-Asialo-GM1 (Poly21460, Biolegend, 35μl/mouse) one day prior and one day after tumor transplantation. For tumor growth experiments, antibody injections were performed every 3-4 days during the course of the experiment.

#### Chemokine neutralization *in vivo*

For neutralization of CCL5 and XCL1, 50μg of anti-CCL5 and 50μg of anti-XCL1 antibodies or of isotype-matched control antibodies were injected i.v. at the time of tumor transplantation, followed by a second injection two days later (R&D Systems; CCL5: AF478 and MAB478; XCL1: AF486, MAB486; Isotype controls: AB-108-C, MAB006).

#### Processing of tumor tissue

Unless stated otherwise, tumors were excised 4 days after transplantation. Mammary tumors from female MMTV-PyMT mice were excised when palpable. Tumor mass of individual tumors was determined using a microscale. For subsequent analysis by flow cytometry, tumors were cut into pieces and digested with Collagenase IV (200U/ml) and DNase I (100μg/ml) for 30min at 37**°**C. Tissue was passed through a 70μm cell strainer (Falcon) and washed with FACS buffer (PBS with 1% FCS and 2mM EDTA) before proceeding with antibody mediated staining. For protein/chemokine analyses, tumors were placed in protein lysis buffer (PBS with Aprotinin, Leupeptin and Pepstatin (all 10μg/ml)) and homogenized using a TissueLyser II (QIAGEN). For RNA isolation, homogenization was performed in RLT buffer (QIAGEN).

#### Chemokine analyses

Total protein content of tumor lysates was quantified by bicinchoninic acid (BCA) assay (Thermo Fisher Scientific). Profiling of intratumoral chemokines was done for 500μg protein from tumor lysates using the Mouse Chemokine Array Kit (R&D Systems) according to the manufacturer’s instructions. Signal was revealed by SuperSignal West Pico Chemiluminescent substrate (Thermo Fisher Scientific); and signal of individual chemokine spots was quantified using ImageJ software. Quantification of chemokines in tumor lysates or from cell culture supernatants was done by flow cytometry using the Cytometric Bead Array (CBA, BD Biosciences). Detection of XCL1 protein in cell culture supernatants was done by ELISA (R&D Systems). Note that the lack of sensitivity of this ELISA precluded analysis of XCL1 protein in tumor extracts.

#### Dendritic cell migration assays

Chemotaxis of cDC1 was analyzed in transwell migration assays. 5x10^5^ DCs were taken up in RPMI/1% BSA and placed into 5μm pore size transwell inserts (Corning), which were placed into wells of a tissue culture plate containing 500μl RPMI/1% BSA ± 100ng/ml CCL5 (R&D) or 150ng/ml XCL1 (R&D). After incubation at 37°C for 2h, cells in the lower compartment were harvested and quantified by flow cytometry. Migration was calculates as % live cells in bottom well relative to input.

#### NK cell stimulation assays

NK cells were purified from spleens or *Ptgs1/Ptgs2*^*−/−*^ BRAF^V600E^ tumors of wild-type C57BL/6J mice by negative selection using the NK Cell Isolation Kit II (Miltenyi Biotec). NK cells were cultured at 37°C in medium containing either 200U/ml recombinant IL-2, 5ng/ml IL-15 or 5ng/ml IL-15:IL-15Rα complexes (Invitrogen) for 16h. To assess chemokine production, NK cells were stimulated with plate-bound anti-NK1.1 antibody (PK136) for 16h. Where indicated, 1-100ng/ml PGE_2_ (Sigma) was added to the *in vitro* culture.

#### Flow cytometry and fluorescence activated cell sorting

Flow cytometric analyses were performed using an LSR Fortessa, LSR Fortessa X20 or FACSymphony (BD Biosciences). Data were analyzed using FlowJo (Tree Star). DAPI (0.5 mg/ml, Sigma-Aldrich) or a Live/Dead fixable cell stain kit (Invitrogen) was used to exclude dead cells in all experiments, and anti-CD16/CD32 antibody (2.4G2) was used to block non-specific binding of antibodies via Fc receptors. The following antibodies were used for flow cytometry: anti-CD3ε (clone 17A2), anti-CD4 (RMA4.5), anti-CD8α (53-6.7), anti-CD8β (53-5.8), anti-CD11b (M1/70), anti-CD11c (N418), anti-CD16/CD32 (2.4G2), anti-CD24 (M1/69), anti-CD45.2 (104), anti-CD49a (HMα1), anti-CD49b (DX5) anti-CD64 (X54-5/7.1) anti-CD103 (M290), anti–Clec9a/DNGR-1 (1F6), anti-CCL5 (2E9/CCL5), anti–MHC class II (MHC II) I-a/I-E (M5/114.15.2), anti-NK1.1 (PK136), anti-PD-1 (29F.1A12), anti-TCRβ (H57-597), anti-TCRγδ (GL3), anti-TIM-3 (RMT3-23) and anti-XCR1 (ZET). Detection of cell death was done using the Annexin V Apoptosis Detection Kit with PI (Biolegend) according to the manufacturer’s instructions. NK cells were identified as live CD45^+^NK1.1^+^CD49b^+^CD3^−^MHCII^−^ cells. CD103^+^ cDC1 were identified as live CD45^+^CD103^+^CD11b^−^CD11c^+^MHCII^+^ cells. Quantification of total cell numbers by flow cytometry was done using fluorescent beads (Beckman Coulter). For intracellular staining of CCL5 *ex vivo*, tumor-bearing mice were injected with brefeldin A (10mg/kg body weight) i.v. and tumors were collected 6h later. Tumor processing was done in presence of brefeldin A (5μg/ml) and cells were fixed in 4% paraformaldehyde for 10min at room temperature. Detection of intracellular mRNA encoding for XCL1 was done by PrimeFlow RNA Assay (Affymetrics and Thermo Fisher Scientific) using a type 1 probe according to the manufacturer’s instructions. Intracellular staining was performed in permeabilization buffer (eBioscience) for 30min and cells were subsequently analyzed by flow cytometry. Staining of intracellular IRF8 (V3GYWCH) or Granzyme B (anti-human, cross-reactive with mouse, clone GB11) was done using the Foxp3/Transcription factor staining buffer set from eBioscience. All antibodies were purchased from Biolegend, BD Biosciences or eBioscience except for anti-DNGR-1, which was produced in house. Sorting of tumor cells after retroviral transduction was done using a BD FACSAria or a BD FACSAria Fusion. Purity of cell populations was determined by reanalysis of a fraction of sorted cell samples.

#### Immunofluorescence Imaging

Tumors were fixed in Antigenfix solution (Diapath). Samples were dehydrated in 30% sucrose prior to embedding in TissueTek OCT freezing medium (Sakura Finetek) and stored at −80 °C. 30μm sections were permeabilized, blocked, and stained in 0.1M Tris (AppliChem) supplemented with 1% BSA, 0.3% Triton X-100 (Gerbu Biotechnik) and normal mouse serum (Life Technologies). Serial tumor sections were prepared and visually inspected by epifluorescence light microscopy before acquisition of representative areas by confocal microscopy. Staining used the following antibodies: anti-CD31 (clone Mec13.3; Biolegend), anti-CD103 (goat polyclonal; R&D Systems), anti-Collagen IV (rabbit polyclonal; Abcam), anti-NK1.1 (PK136; BD Biosciences), anti-MHC class II (MHCII) I-A/I-E (M5/114.15.2; BD Biosciences). Stained sections were mounted in Mowiol and analyzed on a LSM 710 or LSM 780 confocal microscope (Zeiss). Image analysis was performed using Imaris software (Bitplane) on maximum projections of 7-10 Z-plane sections. Semi-automated analyses using the Imaris surface generation tool was used to reconstruct surfaces for CD103^+^ cDC1, CD103^−^MHCII^+^ cells, NK cells and CD31^+^ blood vessels. To calculate the distance of cells to the tumor margin, a surface outlining the margin of the tumor was generated based on Collagen IV staining. Automated quantification of the minimal distances between individual cells, of cells to CD31^+^ blood vessels or the tumor margin was done using the Imaris distance transformation tool.

#### Cloning of retroviral vectors for XCL1 and CCL5 expression

NK cells were isolated from spleen of C57BL/6 WT mice and NK cell mRNA was purified using RNeasy Mini Kit (QIAGEN). Total cDNA was prepared by reverse transcription and DNA was then amplified using the following primers: XCL1-Forward (BamHI) 5′-GGCCCGGGGATCCATGGATGAGACTTCTCCTCCT-3′ and XCL1-Reverse (XhoI) 5′-CGGCCAACCGGCTCGAGTTACCCAGTCAGGGTTA-3′ for XCL1; CCL5-Forward (BamHI) 5′-GGCCCGGGGATCCATGGATGAAGATCTCTGCAG-3′ and CCL5-Reverse (XhoI) 5′-CGGCCAACCGGCTCGAGCTAGCTCATCTCCAAATA-3′ for CCL5. Both PCR products and the target vector pMSCV-IRES-mCherry (Addgene #52114) were then digested with XhoI and BamHI for 1h at 37°C and purified by gel extraction after agarose gel electrophoresis. Ligation was performed for 1h at room temperature using T4 DNA ligase (NEB). The ligation mix was then transformed into One Shot TOP10 chemically competent bacteria (Thermo Fischer Scientific) and plated on ampicillin containing LB-agar plates. Single colonies were then sequenced and used for plasmid isolation using the High speed Maxi Kit (QIAGEN).

#### Retroviral transduction

GP2-293 packaging cells were transfected with a mixture of GeneJuice (Novagen), VSV-G envelope protein-coding plasmid, and a pMSCV-IRES-mCherry plasmid coding for the desired protein or empty as control. On three consecutive days post-transfection, the pseudotyped virus-containing culture medium was harvested, filtered, supplemented with 8 μg/ml polybrene (Sigma-Aldrich), and immediately applied to target cells for spinfection by centrifugation (90min, 2500xg at room temperature). After the incubation, the medium was exchanged for fresh complete RPMI1640 medium. Target cells were passaged at least three times after retroviral transduction and analyzed for mCherry expression as a read out for transduction efficiency. Where necessary, cells were FACS-sorted based on mCherry expression to ensure equal levels of transduction between different cell lines.

#### RNA isolation and quantitative real-time PCR

RNA was isolated using QIAGEN RNeasy Mini Kit and cDNA was synthesized using the Superscript II reverse transcriptase (Invitrogen). Quantitative real-time PCR (qRT-PCR) analysis was performed using Fast SYBR Green Master Mix (Invitrogen) according to the manufacturer’s instructions on an QuantStudio (Thermo Fisher Scientific) using the relative standard curve method. The PCR conditions were 2min at 50°C, 10min at 95°C followed by 40 2-step cycles of 15 s at 95°C and 1 min at 60°C. Primers for XCL1 (Xcl1-Forward 5′-CTTTCCTGGGAGTCTGCTGC-3′ and Xcl1-Reverse 5′-CAGCCGCTGGGTTTGTAAGT-3′) and HPRT (HPRT-Forward 5′-TCAGTCAACGGGGGACATAAA-3′ and HPRT-Reverse 5′-GGGGCTGTACTGCT-3′ TAACCAG) as normalization control were used to assess relative gene expression.

#### Analysis of gene expression data

Publically available datasets were downloaded from gene expression omnibus (GEO, https://www.ncbi.nlm.nih.gov/geo/). Affymetrix arrays were normalized using RMA. Illumina arrays were quantile normalized and log2 transformed. All data from expression arrays were processed within R. Data from the following gene expression datasets were used in this paper: GSE15907, GSE24759, GSE77671. For analyses of chemokines expressed by NK cells (GSE15907), analyses were restricted to probes with expression values above the standard post-normalization threshold of 120 indicating expression above background.

#### Bioinformatic analysis of cancer patient data

RSEM normalized expression datasets from The Cancer Genome Atlas (TCGA) were downloaded from Firehose (https://gdac.broadinstitute.org/). Hierarchical clustering of expression data was plotted as heatmaps using the ‘gplots’ package (version 3.0.1), where red indicates higher and blue indicates lower expression relative to the mean expression per gene. For generation of gene expression signatures, normalized expression values were log2-transformed and ranked by the mean expression value of signature genes. The following gene signatures were used: chemokines (*XCL1*, *XCL2*, *CCL5),* NK cells (*NCR1*, *NCR3*, *KLRB1*, *CD160*, *PRF1*), cDC1 (*CLEC9A*, *XCR1*, *CLNK*, *BATF3*) and CD8 T cells (*CD8A*, *CD8B*, *CD3E*). Overall survival analyses were performed for the top and bottom quartile expression ranked values for selected genes or the ranked sum expression of gene signature and plotted for Kaplan-Meier curves using GraphPad Prism (GraphPad). Gene signature analyses for another cohort of breast cancer (including TNBC) patients were done using the KM plotter software (http://kmplot.com) (Szász et al., 2016).

### Quantification and Statistical Analysis

#### Statistical analysis

All statistical analyses were performed using GraphPad Prism software (GraphPad). Statistical significance was determined using an unpaired two-tailed Student’s t test. Statistical analyses for three or more groups and tumor growth profiles were done by ANOVA. Correlation analyses were performed using Pearson correlation. The log-rank (Mantel-Cox) test was used to determine statistical significance for overall survival in cancer patient data from TCGA. Data are shown as mean ± SD or mean ± SEM as indicated in the figure legends. Significance was assumed with ^∗^p < 0.05; ^∗∗^p < 0.01; ^∗∗∗^p < 0.001.
