# Supplementary material for: NK Cells Stimulate Recruitment of cDC1 into the Tumor Microenvironment Promoting Cancer Immune Control
Source: Cell. 2018 Feb 22;172(5):1022–1037.e14. doi: 10.1016/j.cell.2018.01.004 (PMC5847168; doi:10.1016/j.cell.2018.01.004)
Supplement: Document S1. Table S1 [file mmc1.pdf]

Cell, Volume *172*

Supplemental Information

NK Cells Stimulate Recruitment of cDC1 into the Tumor Microenvironment Promoting Cancer Immune Control

Jan P. Böttcher, Eduardo Bonavita, Probir Chakravarty, Hanna Blees, Mar Cabeza-Cabrerizo, Stefano Sammicheli, Neil C. Rogers, Erik Sahai, Santiago Zelenay, and Caetano Reis e Sousa

	cDC1 signature		NK cell signature		CLEC9A	
	HR [95% CI]	Median survival ratio [95% CI]	HR [95% CI]	Median survival ratio [95% CI]	HR [95% CI]	Median survival ratio [95% CI]
SKCM	0.5395 [0.3706 - 0.7853]	1.93 [1.323 – 2.815]	0.4874 [0.3292 - 0.7216]	2.549 [1.727 – 3.761]	0.3987 [0.2714 – 0.5855]	3.789 [2.584 – 5.558]
BRCA	0.561 [0.3691 - 0.8526]	1.125 [0.7323- 1.728]	0.6006 [0.3935 - 0.9165]	0.9821 [0.6342 - 1.521]	0.6168 [0.4006 – 0.9499]	1.125 [0.7275 – 1.739]
HNSC	0.5077 [0.3439 - 0.7496]	1.68 [1.137 - 2.483]	0.6198 [0.432 - 0.8892]	1.983 [1.379 - 2.851]	0.4881 [0.3305 – 0.721]	1.68 [1.125 – 2.51]
LUAD	0.4867 [0.3226 - 0.7341]	1.693 [1.098 - 2.612]	0.5776 [0.3855 - 0.8654]	1.533 [1.006 - 2.334]	0.6206 [0.4143 – 0.9298]	1.412 [0.9406 – 2.119]

Table S1. Related to Figure 7. Prognostic value of cDC1-specific genes and NK cell specific genes for survival of human cancer patients.

Patient data were ordered according to the sum ranked expression levels gene signatures containing cDC1, NK cell specific transcripts or the transcript levels of *CLEC9A*. Top and bottom quartiles of ordered patient data were compared for overall survival. Hazard ratio (HR) and Median survival ratio for top/bottom quartiles are shown. CI: confidence interval.
